# Amyloid and tau may moderate practice effects in semantic and episodic memory in a cognitively unimpaired at-risk sample

**DOI:** 10.1093/braincomms/fcaf390

**Published:** 2025-10-09

**Authors:** Kristin Basche, Madeline Hale, Erin Jonaitis, Tobey J Betthauser, Bradley T Christian, Bruce P Hermann, Sterling C Johnson, Kimberly Mueller, Rebecca Langhough

**Affiliations:** Department of Medicine, Division of Geriatrics and Gerontology, School of Medicine and Public Health, University of Wisconsin-Madison, Madison, WI 53705, USA; Department of Communication Sciences and Disorders, University of Wisconsin-Madison, Madison, WI 53706, USA; Department of Medicine, Division of Geriatrics and Gerontology, School of Medicine and Public Health, University of Wisconsin-Madison, Madison, WI 53705, USA; Wisconsin Alzheimer's Institute, School of Medicine and Public Health, University of Wisconsin-Madison, Madison, WI 53705, USA; Wisconsin Alzheimer's Disease Research Center, School of Medicine and Public Health, University of Wisconsin-Madison, Madison, WI 53705, USA; Department of Medicine, Division of Geriatrics and Gerontology, School of Medicine and Public Health, University of Wisconsin-Madison, Madison, WI 53705, USA; Wisconsin Alzheimer's Institute, School of Medicine and Public Health, University of Wisconsin-Madison, Madison, WI 53705, USA; Wisconsin Alzheimer's Disease Research Center, School of Medicine and Public Health, University of Wisconsin-Madison, Madison, WI 53705, USA; Wisconsin Alzheimer's Disease Research Center, School of Medicine and Public Health, University of Wisconsin-Madison, Madison, WI 53705, USA; Department of Medical Physics, University of Wisconsin-Madison School of Medicine and Public Health, Madison, WI 53705, USA; Wisconsin Alzheimer's Institute, School of Medicine and Public Health, University of Wisconsin-Madison, Madison, WI 53705, USA; Department of Neurology, University of Wisconsin-Madison School of Medicine and Public Health, Madison, WI 53705, USA; Department of Medicine, Division of Geriatrics and Gerontology, School of Medicine and Public Health, University of Wisconsin-Madison, Madison, WI 53705, USA; Wisconsin Alzheimer's Institute, School of Medicine and Public Health, University of Wisconsin-Madison, Madison, WI 53705, USA; Wisconsin Alzheimer's Disease Research Center, School of Medicine and Public Health, University of Wisconsin-Madison, Madison, WI 53705, USA; Department of Communication Sciences and Disorders, University of Wisconsin-Madison, Madison, WI 53706, USA; Wisconsin Alzheimer's Institute, School of Medicine and Public Health, University of Wisconsin-Madison, Madison, WI 53705, USA; Wisconsin Alzheimer's Disease Research Center, School of Medicine and Public Health, University of Wisconsin-Madison, Madison, WI 53705, USA; Department of Medicine, Division of Geriatrics and Gerontology, School of Medicine and Public Health, University of Wisconsin-Madison, Madison, WI 53705, USA; Wisconsin Alzheimer's Institute, School of Medicine and Public Health, University of Wisconsin-Madison, Madison, WI 53705, USA; Wisconsin Alzheimer's Disease Research Center, School of Medicine and Public Health, University of Wisconsin-Madison, Madison, WI 53705, USA

**Keywords:** Alzheimer’s disease, PET biomarkers, Logical memory, practice effects

## Abstract

Previous studies in pre-clinical Alzheimer’s disease have found associations between amyloid and practice effects. Additionally, studies within our research group have found associations between practice effects and cognitive tests, including language-based measures, as well as associations between language-based measures and Alzheimer’s disease biomarkers. The purpose of this study is to bridge the gaps between these areas to further understand how practice effects may or may not explain additional variance between biomarker groups and to expand on current literature by incorporating tau status into these models. Our study had three main aims: (1) determine which of our proposed operationalizations of practice effects performed best, (2) explore the impact of amyloid on practice effects and (3) explore the impact of combined amyloid and tau status on practice effects for language-based measures: the proper names composite from the Logical Memory story recall task, as well as the total score, animal fluency, and letter fluency tasks. Participants from the Wisconsin Registry for Alzheimer’s Prevention study with amyloid positron emission tomography (PET) scans and item-level Logical Memory data were included in these analyses (*n* = 442); the Aim 3 subset, requiring both amyloid and tau PET scans, included *n* = 397. Linear mixed effects models were used to explore our aims; for Aim 1 we utilized Akaike information criteria (AIC) to determine which operationalization performed best and for Aims 2 and 3 we used an interaction of biomarker * practice to determine if biomarker status(es) moderated the impact of practice on our measures of interest. Comparing the base model AIC to the models including practice showed that inclusion improved model fit for all outcomes Proper names from Logical Memory and the total score of Logical Memory showed a moderating effect of amyloid status on practice effects. Sensitivity analyses indicated that it may be age that is driving the association, however. A similar pattern was seen upon testing amyloid/tau (A/T) status and practice effect moderation on our outcomes, such that participants who were A+T+ did not appear to benefit from practice as much as the A−T− participants did. Future studies should seek to tease apart the intertwined impacts of age, practice and cognitive decline.

## Teaser

In this article, Basche *et al*. explore the potential utility of proper name recall and practice effects as indicators of preclinical Alzheimer’s disease. Using linear mixed effects models, they show that practice effects may be sensitive to PET biomarker status in the Logical Memory total and proper name recall scores.

## Graphical Abstract

**Graphical Abstract:**
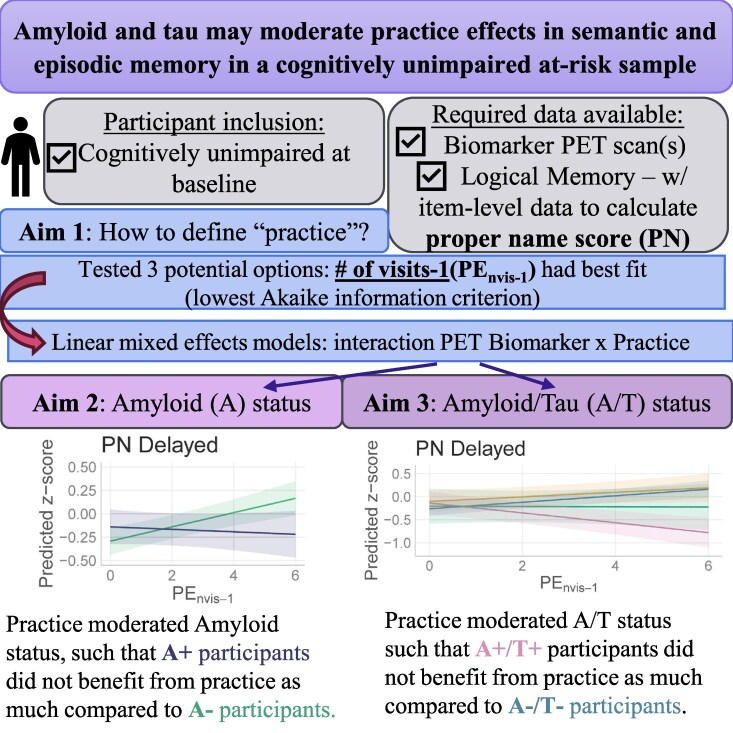

## Introduction

Alzheimer’s disease (AD) is a neurodegenerative condition marked by the accumulation of beta-amyloid (Aβ) plaques and tau neurofibrillary tangles (T), leading to neuronal death and cognitive decline.^1,2^ These pathological changes often begin decades before the clinical onset of symptoms, underscoring the need for early detection methods to intervene during the preclinical phase of AD.^3,4^ Early interventions at this stage have the greatest potential to modify disease progression, making the identification of sensitive cognitive markers crucial.^5^

The National Institute on Aging-Alzheimer’s Association (NIA-AA) framework categorizes cognitively unimpaired individuals with amyloid-β positivity as being in the early stage of AD pathological change or preclinical AD.^2^ These individuals represent an ideal cohort for studying early cognitive markers of AD. Among various approaches, the study of proper name recall and practice effects (PE) hold promise.

Proper name recall, a semantic and episodic memory-based measure, has shown particular sensitivity to AD pathology. Unlike common nouns, proper names are unique, in that they do not typically carry semantic information that links them to a category or concept, potentially carrying a higher cognitive load due to less redundant information.^6,7^ Such difficulties in retrieval of proper names, in comparison to regular nouns, has been shown to be accentuated in cases of probable AD.^8^ Furthermore, studies from our group have linked deficits in proper name recall to amyloid and tau burden, suggesting its utility as an early marker of AD.^9,10^ The observed impairment in proper name retrieval in AD aligns with early pathological changes in the anterior temporal lobe and parahippocampal regions, which are critical for recall and long-term memory consolidation.^11,12^

Another potential marker of early decline, PE refers to improved performance on cognitive tasks due to repeated exposure, which can mask cognitive decline in longitudinal studies.^13^ However, reduced PE may signal underlying age-related or pathological changes, offering a potential early indicator of AD-related accelerated long-term forgetting (ALF).

ALF describes the phenomenon where information initially encoded and retained over short intervals is forgotten more rapidly over extended periods.^14^ This process may reflect hippocampal atrophy and medial temporal lobe dysfunction—hallmarks of early AD.^1,15^ By leveraging PE as a proxy for ALF, researchers may better detect subtle cognitive changes indicative of preclinical AD.

Research has linked greater amyloid burden to diminished PE on memory tests.^16,17^ However, studies have varied in their methodologies and focus predominantly on short-term intervals, with limited exploration of PE across different cognitive measures and over longer periods. For example, Duff and colleagues^17^ used a one-week interval to measure PE with the Brief Visuospatial Memory Test-Revised (BVMT-R), while others have employed intervals ranging from days to several weeks.^18,19^ While most studies investigating PE use short-term practice, these methods do not coincide with the typical timeline in which cognitive testing typically occurs and it is unclear if PE differ by these variations in time. Longitudinal studies have also shown similar results of reduced PE.^20-23^ These longitudinal methodologies were similarly utilized to explore PE within the Wisconsin Registry for Alzheimer’s Prevention (WRAP) cohort and have also demonstrated significant PE across cognitive domains including memory, working memory and speed and flexibility.^24,25^

Due to the novelty of proper name recall and our hypothesis of this being a particularly sensitive measure, we also considered the Logical Memory total score to compare the proper name recall composites with the total score. In our analyses, we used both the immediate and delayed recall scores from Logical Memory. The immediate recall scores potentially capture verbal learning, while the delayed recall captures episodic memory.^26^ Additionally, we selected several language-based tasks from the WRAP neuropsychological battery for the present study, considering tasks that were likely to elicit PE, including letter fluency (CFL) and Animal Naming tasks, which previous studies have demonstrated as having diminished or absent PE in groups with cognitive decline along the AD continuum.^27^ Thus in this study, we chose to investigate proper names, category fluency, and letter fluency as measures of semantic, lexical and phonological processing, with the latter tests reflecting standardized measures and the proper names composite representing a more experimental process score.

Given the established links between proper name recall and amyloid biomarkers, as well as the links between amyloid biomarkers and PE, we sought to investigate the relationship between proper name recall and PE. Given the complexity of proper name recall, we sought to determine if proper name recall was also subject to differential PE in AD biomarker groups. This study aims to build on prior WRAP findings by:

1. Comparing different methods of quantifying PE to determine their efficacy in explaining cognitive variability before accounting for biomarker status.
2. Assessing whether amyloid positron emission tomography (PET) status moderates the relationship between PE and novel semantic memory and traditional cognitive measures using linear mixed-effects models.
3. Exploring the combined effects of amyloid and tau PET statuses on PE of novel and traditional measures, with subgroup analyses for A−/T−, A−/T+, A+/T− and A+/T+ classifications.

These aims will help elucidate the role of PE among novel and traditional cognitive measures as an early cognitive marker, advancing our understanding of preclinical AD and informing intervention strategies.

## Materials and methods

### Participants

Participants were recruited from the WRAP study, an ongoing longitudinal cohort study consisting of a sample of cognitively unimpaired, middle-aged adults enriched for a family history of AD.^28^ Recruitment for WRAP began in 2001 and requires that participants do not have a prior diagnosis of dementia or evidence of dementia upon baseline assessment. The data from these analyses were drawn from the May 2023 semi-annual data freeze. At that time, *n* = 1752 had completed at least a baseline assessment (median = 3 visits, range = 1–8). Study visits consist of detailed neuropsychological testing, medical examinations and health and lifestyle questionnaires, which are all completed on a biennial schedule. Participants are recruited predominantly from the upper Midwest, with testing sites in Madison, LaCrosse and Milwaukee.

**Figure 1:**
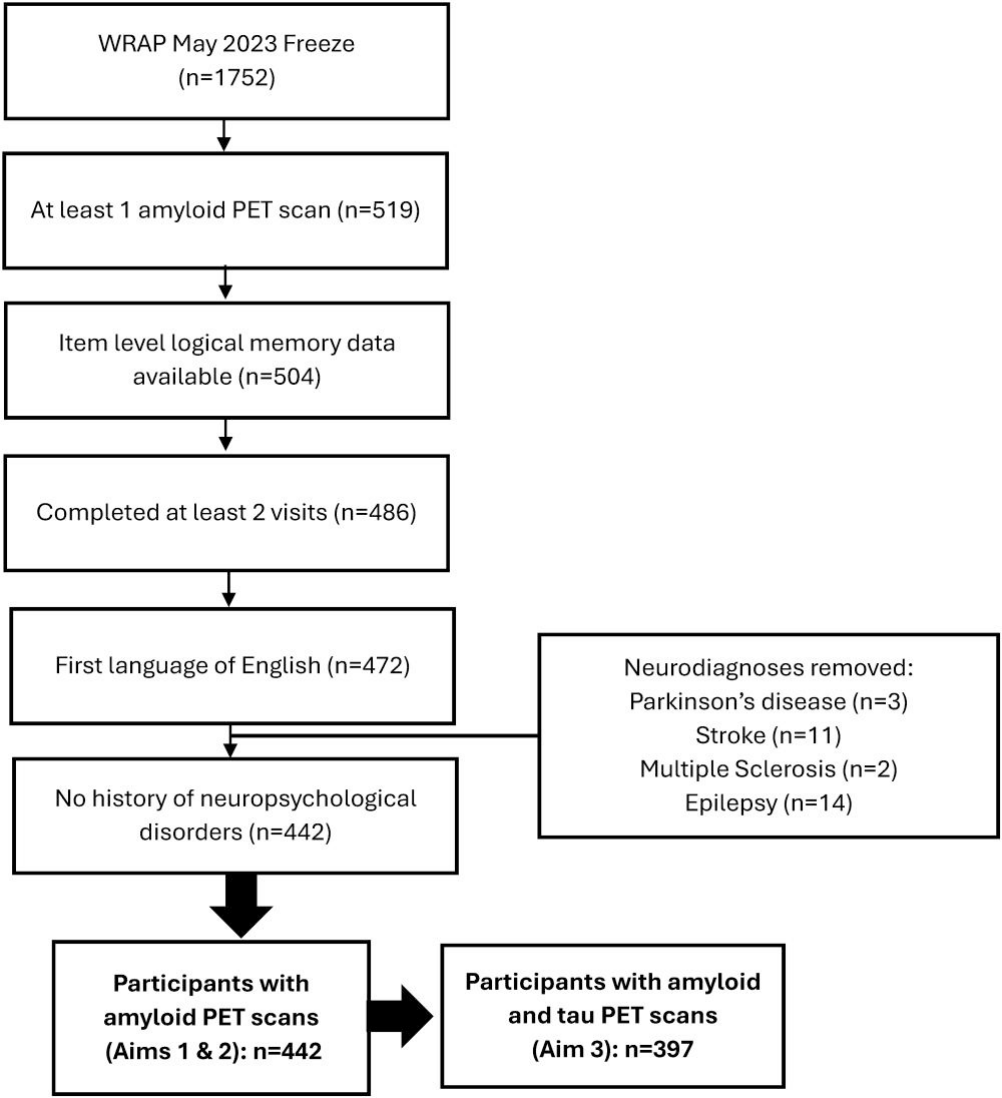
**Consort diagram of inclusion criteria.** Consort diagram illustrating how we determined the samples for our analyses. WRAP = Wisconsin Registry for Alzheimer’s Prevention.

Participants from the WRAP study were included in the present study if they had completed language tasks for at least two visits (letter fluency, category fluency and Logical Memory story recall) and had at least one C-Pittsburgh Compound-B (PiB) amyloid PET scan completed. Participants were excluded from the sample if they were impaired at their baseline visit, their first language was not English, or if they had history of neurological disorders, including Parkinson’s disease, multiple sclerosis, stroke, epilepsy or seizures (Fig. 1). For our exploratory Aim 3 analyses, we included a subset of participants with MK-6240 PET imaging to explore the added association of tau (*n* = 397) and PE on language outcomes.

### Neuropsychological testing

Our primary variable of interest in this study was proper name recall, a theoretical composite that potentially captures semantic memory. We used item-level data from the Wechsler Memory Scale-Revised, Logical Memory^29^ to calculate the immediate and delayed recall from Stories A & B of the proper name scores^10^ (0–9 points possible for each condition). We also considered the Logical Memory total score (0–50 points possible for each, immediate and delayed) to compare the proper names outcome performance with the total score. We also included the CFL and Animal Naming scores as measures of fluency.

### Neuroimaging

Participants were selected who underwent a 70-min dynamic [^11^C]PiBscan (PiB) on a Siemens EXACT HR+ scanner [and prior to 2015, a T1-weighted magnetic resonance imaginig (MRI) scan on a GE 3.0 T MR750 using an 8-channel headcoil] at the University of Wisconsin-Madison Waisman Center Brain Imaging Lab. For a complete description of the PiB protocol, including, radiosynthesis, acquisition and reconstruction parameters, image processing and quantification please refer to the previous paper by Johnson and colleagues.^30^ PiB PET status (A+/−) was defined using a previously derived positivity threshold of global distribution volume ratio (DVR) > 1.19.^31,32^ WRAP participants that completed amyloid imaging were then invited for a separate scan for tau PET using a 70 to 110-min protocol with [F18]MK6420. For a full description of the tau PET imaging protocol please refer to Betthauser and colleagues.^33^ Tau PET status (T+/−) was determined by setting the MK-6240 standard uptake value ratio positivity threshold at two standard deviations above the mean of the A− group in the entorhinal cortex^34^ (MK-6240 SUVR > 1.27).

### Practice effects

In order to assess PE in our models, we tested several iterations of a variable to elucidate the number of previous exposures to a task. This concept has also been referred to as ‘retest gains’ in other publications,^35^ which is a distinct variable from longitudinal change over time and is ultimately how we defined practice. We defined practice in each of the following ways: number of cognitive task exposures—1 (‘PE_nvis-1_’, median = 2, min = 0, max = 6), the square root of PE_nvis-1_ (‘PE_sqrt_’), and a categorical measure (‘PE_cat_’); with PE_cat_ coded such that 0 = first task exposure, 1 = 1 previous exposure, 2 = 2 previous exposures, and 3 = 3 or more previous task exposures. PE_sqrt_ and PE_cat_ were designed as a means to incorporate the diminishing returns effect that has been previously reported, such that after the third exposure to a task, the benefits of practice begin to diminish.^36^ It is important to note that we selected these means of defining practice in order to separate it from the time that has passed between test intervals, which is included in the model as participants’ age at testing. We also avoided using change-score models because we did not want to ascribe the differences in task performance to practice alone, as other factors can impact participants’ performance outside of practice (i.e. cognitive decline, poor performance due to distractions or illness, etc.).

### Statistical methods

All analyses were run using R (Version 4.4.0). We used linear mixed effects models (lme4 package^37^) to address each aim, we decided upon these models to avoid noted problems in change-score based methods of longitudinal PE as described by Machulda and colleagues.^38^ Preliminary analyses began with determining the appropriate random effects and age fixed effects to include. Specifically, for each language outcome we ran models with a random intercept alone and with a random age slope to see which model had the optimal Akaike information criteria (AIC). Once we had our random effects, we added linear and quadratic fixed effects for age (mean-centred around 63.13), again using AIC for model selection. To the selected age model for each outcome, we added known covariates of sex and Wide Range Achievement Test (WRAT)-III reading scores—this fit constituted the base model for Aim 1. We then compared this base model to three other models including different PE operationalizations described in the previous section. AIC were used to determine whether PE were present, and if so, which operationalization was best for each of the cognitive measures (proper names immediate and delayed recall, Logical Memory total scores for immediate and delayed recall, letter fluency, category fluency).

After determining the PE operationalization of best fit, we ran all subsequent models using the PE_nvis-1_ operationalization and incorporated the same base model and the AD biomarker * PE interactions. Model results without the biomarker interaction are included in Supplementary Table 1 to demonstrate PE prior to inclusion of interaction terms, showing positive slopes for practice across all tests. Specifically, Aim 2 models added amyloid status * PE interaction(s) while Aim 3 models added A/T status * PE interactions (A−T− = reference group). Interaction terms that failed to reach significance levels below *P* = 0.05 thresholds were removed and models were rerun prior to interpreting PE and biomarker status associations with longitudinal outcomes. When significant interactions were present, simple slopes and their confidence intervals were described and pairwise comparisons were made using the emmeans R package.^39^ Sensitivity analyses added the corresponding AD biomarker status * age interaction to each model.

## Results

### Participant characteristics

Participant (*n* = 442) characteristics are displayed in Table 1 overall (Aim 1) and by amyloid status (Aim 2). For these analyses, the majority of participants were non-Hispanic white (*n* = 420, 95.0%), female (*n* = 301, 68.1%), and had a mean baseline age of 57.8 (SD = 6.73). Of this sample, 116 (26.2%) were A+. Participants differed by their amyloid status on baseline neuropsychological testing visit age, family history of AD, APOE ε4 allele carriage, and the age at most recent amyloid PET scan (*P* < 0.01), such that A+ participants tended to be older, carried at least one APOE ε4 allele, and were positive for family history of AD.

**Table 1:** Sample characteristics—amyloid status

	Overall (*n* = 442)	A− (*n* = 326, 73.8%)	A+ (*n* = 116, 26.2%)	*P*-value
Baseline NP age, mean (SD)	57.8 (6.67)	57.1 (6.96)	59.7 (5.37)	**<0**.**01**
Sex, female, *n* (%)	301 (68.1%)	217 (66.6%)	84 (72.4%)	0.30
Race, *n* (%)				0.33
Native American or American Indian	7 (1.6%)	5 (1.5%)	2 (1.7%)	
Black or African American	15 (3.4%)	12 (3.7%)	3 (2.6%)	
White	420 (95.0%)	309 (94.8%)	111 (95.7%)	
Family History, Positive, *n* (%)	329 (74.4%)	230 (70.6%)	99 (85.3%)	**<0**.**01**
APOE ℇ4 alleles, 1+ alleles, *n* (%)	164 (37.1%)	88 (27.0%)	76 (65.5%)	**<0**.**01**
WRAT-III reading score, mean (SD)	106 (9.32)	106 (9.25)	107 (9.56)	0.63
Test–retest intervals (years), mean (SD)	2.61 (0.47)	2.60 (0.48)	2.65 (0.49)	0.42
Number of follow-ups, mean (SD)	4.97 (1.32)	4.90 (1.32)	5.16 (1.30)	0.07
Follow-up duration (years), Mean (SD)	10.3 (3.51)	10.1 (3.52)	10.8 (3.43)	**0**.**04**
Most recent diagnosis, *n* (%)				**<0**.**01**
Cognitively unimpaired	409 (92.6%)	318 (97.5%)	91 (78.4%)	
MCI	23 (5.2%)	7 (2.1%)	16 (13.8%)	
Impaired (not MCI)	2 (0.5%)	0 (0%)	2 (1.7%)	
Dementia	8 (1.8%)	1 (0.3%)	7 (6.0%)	
Baseline CFL, mean (SD)	47.1 (10.9)	46.4 (10.9)	49.0 (10.9)	**0**.**03**
Most Recent CFL, mean (SD)	50 (11.8)	49.6 (11.8)	50.9 (11.6)	**0**.**34**
Baseline Animal Naming, mean (SD)	22.9 (5.41)	22.9 (5.20)	23.0 (6.00)	0.96
Most recent Animal Naming, mean (SD)	22.2 (5.22)	22.3 (5.09)	21.8 (5.57)	0.31
Baseline LM Total Immediate, mean (SD)	29.6 (6.51)	29.4 (6.40)	30.2 (6.81)	0.31
Most recent LM Total Immediate, mean (SD)	27.7 (7.18)	28.3 (6.45)	26.1 (8.74)	**0**.**01**
Baseline LM Total Delayed, mean (SD)	26.2 (7.14)	25.9 (7.05)	27.1 (7.36)	0.15
Most recent LM Total Delayed, Mean (SD)	25.0 (8.27)	25.6 (7.27)	23.2 (10.4)	**0**.**02**
Baseline PN Immediate, mean (SD)	6.35 (1.63)	6.40 (1.64)	6.22 (1.60)	0.33
Most recent PN Immediate, mean (SD)	5.76 (1.82)	5.95 (1.67)	5.25 (2.11)	**<0**.**01**
Baseline PN Delayed, mean (SD)	4.91 (2.13)	4.93 (2.10)	4.85 (2.22)	0.75
Most recent PN Delayed, mean (SD)	4.34 (2.26)	4.56 (2.10)	3.74 (2.56)	**<0**.**01**
Age at most recent PET scan, mean (SD)	68.8 (7.19)	67.8 (7.32)	71.7 (5.96)	**<0**.**01**

Participant characteristics by amyloid status, includes all participants in Aims 1 and 2 analyses. PiB PET status (A+/−) was defined using a previously derived positivity threshold of global DVR > 1.19. A+ = elevated amyloid, A− = non-elevated amyloid. NP = neuropsychological testing visit. LM, Logical Memory; PN, Proper Name recall (derived from Logical Memory test). Test–retest interval corresponds to the average time between each visit, number of follow-ups refers to the number of visits each participant completed, and follow-up duration refers to the length of time from baseline to most recent visit. Chi-square tests were used to assess the differences for categorical variables and t-tests were used for continuous variables. Bolded *P*-values meeting significance threshold of *P* < 0.05.

Participant characteristics overall and by A/T status are depicted in Table 2 (Aim 3). For these analyses, there were *n* = 397 participants with both amyloid and tau PET scans, the majority of whom were non-elevated for both amyloid and tau [A−T−, *n*(%) = 266 (67.0%)]. Participants differed by their amyloid status on baseline neuropsychological testing visit age, family history of AD, APOE ε4 allele carriage and the age at most recent amyloid and tau PET scans (*P* < 0.01).

**Table 2:** Sample characteristics by A/T status

	Overall (*n* = 397)	A−T− (*n* = 266, 67.0%)	A−T+ (*n* = 20, 5.0%)	A+T− (*n* = 57, 14.4%)	A+T+ (*n* = 54, 13.6%)	*P*-value
Baseline NP age, Mean (SD)	57.8 (6.68)	56.7 (6.91)	62.2 (6.30)	59.4 (5.35)	60.0 (5.44)	**<0**.**01**
Sex, female, *n* (%)	267 (67.3%)	172 (64.7%)	16 (80.0%)	38 (66.7%)	41 (75.9%)	0.25
Race, *n* (%)						0.98
Native American or American Indian	376 (94.7%)	251 (94.4%)	19 (95.0%)	55 (96.5%)	51 (94.4%)	
Black or African American	15 (3.8%)	11 (4.1%)	1 (5.0%)	1 (1.8%)	2 (3.7%)	
White	6 (1.5%)	4 (1.5%)	0 (0%)	1 (1.8%)	1 (1.9%)	
APOE ℇ4 1+ alleles, n (%)	150 (37.8%)	70 (26.3%)	8 (40.0%)	34 (59.6%)	38 (70.4%)	**<0**.**01**
Family history, positive, *n* (%)	293 (73.8%)	181 (68.0%)	18 (90.0%)	44 (77.2%)	50 (92.6%)	**<0**.**01**
WRAT-III reading score, mean (SD)	106 (9.37)	106 (9.41)	109 (7.70)	106 (11.2)	107 (7.54)	0.22
Test–Retest intervals (years), mean (SD)	2.62 (0.48)	2.61 (0.48)	2.59 (0.30)	2.61 (0.53)	2.69 (0.47)	0.64
Number of follow-ups, mean (SD)	5.05 (1.29)	5.00 (1.30)	5.00 (1.30)	5.25 (1.27)	5.13 (1.26)	0.56
Follow-up duration (years), mean (SD)	10.5 (3.40)	10.3 (3.37)	10.5 (3.99)	10.9 (3.19)	11.0 (3.54)	0.26
Most recent diagnosis, *n* (%)						**<0**.**01**
Cognitively unimpaired	370 (93.2%)	263 (98.9%)	19 (95.0%)	50 (87.7%)	38 (70.4%)	
MCI	19 (4.8%)	3 (1.1%)	1 (5.0%)	5 (8.8%)	10 (18.5%)	
Impaired (not MCI)	2 (0.5%)	0 (0%)	0 (0%)	2 (3.5%)	0 (0%)	
Dementia	6 (1.5%)	0 (0%)	0 (0%)	0 (0%)	6 (11.1%)	
Baseline CFL, mean (SD)	47.2 (10.9)	46.5 (11.2)	47.0 (10.2)	47.3 (10.6)	50.9 (9.52)	0.10
Most recent CFL, mean (SD)	50.2 (11.9)	50.0 (12.1)	47.1 (10.8)	52.1 (13.0)	49.8 (9.79)	0.41
Baseline Animal Naming, mean (SD)	23.1 (5.42)	23.2 (5.28)	21.3 (3.06)	23.8 (6.68)	22.1 (5.15)	0.08
Most recent Animal Naming, mean (SD)	22.3 (5.19)	22.6 (5.06)	20.5 (4.06)	23.8 (5.46)	19.7 (4.97)	**<0**.**01**
Baseline LM Total Immediate, mean (SD)	29.6 (6.48)	29.4 (6.41)	29.7 (4.78)	30.6 (6.79)	29.7 (4.78)	0.41
Most recent LM Total immediate, mean (SD)	27.7 (7.11)	28.5 (6.30)	26.1 (5.71)	28.6 (7.95)	23.2 (8.68)	**<0**.**01**
Baseline LM Total Delayed, mean (SD)	26.3 (7.05)	26.1 (6.96)	25.6 (5.87)	27.1 (7.75)	25.6 (5.87)	0.68
Most recent LM Total Delayed, mean (SD)	25.1 (8.11)	26.1 (7.04)	23.2 (5.80)	26.5 (8.86)	19.8 (10.6)	**<0**.**01**
Baseline PN Immediate, mean (SD)	6.36 (1.65)	6.44 (1.67)	6.20 (1.64)	6.14 (1.61)	6.28 (1.65)	0.33
Most recent PN Immediate, mean (SD)	5.72 (1.82)	5.98 (1.63)	5.32 (1.80)	5.72 (2.02)	4.65 (2.09)	**<0**.**01**
Baseline PN Delayed, mean (SD)	4.91 (2.14)	4.95 (2.12)	4.75 (1.92)	4.93 (2.19)	4.74 (2.87)	0.50
Most recent PN Delayed, mean (SD)	4.36 (2.24)	4.68 (2.06)	3.84 (1.95)	4.51 (2.29)	2.90 (2.50)	**<0**.**01**
Age at most recent amyloid PET scan, mean (SD)	69.4 (6.96)	68.1 (7.02)	73.6 (6.30)	71.2 (5.48)	72.5 (6.35)	**<0**.**01**
Age at most recent tau PET scan, mean (SD)	69.3 (7.00)	68.0 (7.04)	73.5 (6.19)	71.1 (5.61)	72.5 (6.40)	**<0**.**01**

Participant characteristics by A/T status, includes all participants in Aim 3 analyses. PiB PET status (A+/−) was defined using a previously derived positivity threshold of global DVR > 1.19. Tau PET status (T+/−) was determined by setting the MK-6240 standard uptake value ratio positivity threshold at two standard deviations above the mean of the A− group in the entorhinal cortex (MK-6240 > 1.27). A+ = elevated amyloid, A− = non-elevated amyloid, T+ = elevated tau, T− = non-elevated tau. NP, neuropsychological testing visit; LM, Logical Memory; PN, Proper Name recall (derived from Logical Memory test). Test–retest interval corresponds to the average time between each visit, number of follow-ups refers to the number of visits each participant completed, and follow-up duration refers to the length of time from baseline to most recent visit. Chi-square tests were used to assess the differences for categorical variables and ANOVAs were used for continuous variables. Bolded *P*-values meeting significance threshold of *P* < 0.05.

### Aim 1: comparing PE operationalizations

Aim 1 final models are presented in Table 3. Comparing the base model AIC to the models including practice showed that inclusion of PE improved model fit for all outcomes. PE_nvis-1_ tended to perform the best or equivalent to the other operationalizations (ΔAIC best-PE_nvis-1_: Animal Naming = 0.41, proper names − immediate = 2.46), and as such was used as the operationalization of PE in all subsequent analyses.

**Table 3:** Comparisons of practice effects model building

	PN—Immediate	PN—Delayed	LM—Total score immediate	LM—Total score delayed	Animal Naming	Letter fluency (CFL)
Base model	5289.262	5117.64	4791.94	4714.12	3952.54	4287.36
PE_nvis-1_	5282.96	**5112**.**50**	**4780**.**16**	**4674**.**94**	3943.85	**4243**.**61**
PE_sqrt_	**5280**.**50**	5113.99	4786.47	4688.88	**3943**.**44**	4251.02
PE_cat_	5283.94	5113.45	4780.70	4679.48	3946.78	4257.37
(PE_nvis-1_–PE_sqrt_)		1.49	6.31	13.94		7.41
(PE_nvis-1_–PE_cat_)		0.95	0.54	4.54		13.76
(PE_sqrt_–PE_nvis-1_)	2.46				0.41	
(PE_sqrt_–PE_cat_)	3.44				3.351	

Base model: outcome ∼ age (centred) + gender + WRAT-III Standard Reading Score + random effects [+ quadratic age for CFL and LM Immediate and Delayed]. Random effects were limited to random intercept only for Animal Naming, CFL, and LM Delayed; random slope and intercept was included for LM Immediate, PN Immediate, and PN Delayed outcomes. The model with the lowest AIC for each task is bolded. Comparisons between change in AIC (best-other) included, a change <2 indicates models are too similar to determine a ‘best’ model. LM = Logical Memory, PN = Proper Name recall. Bolded values for lowest AIC (i.e. optimal model) for each outcome.

### Aim 2: PExAmyloid Status—proper name recall

**Figure 2:**
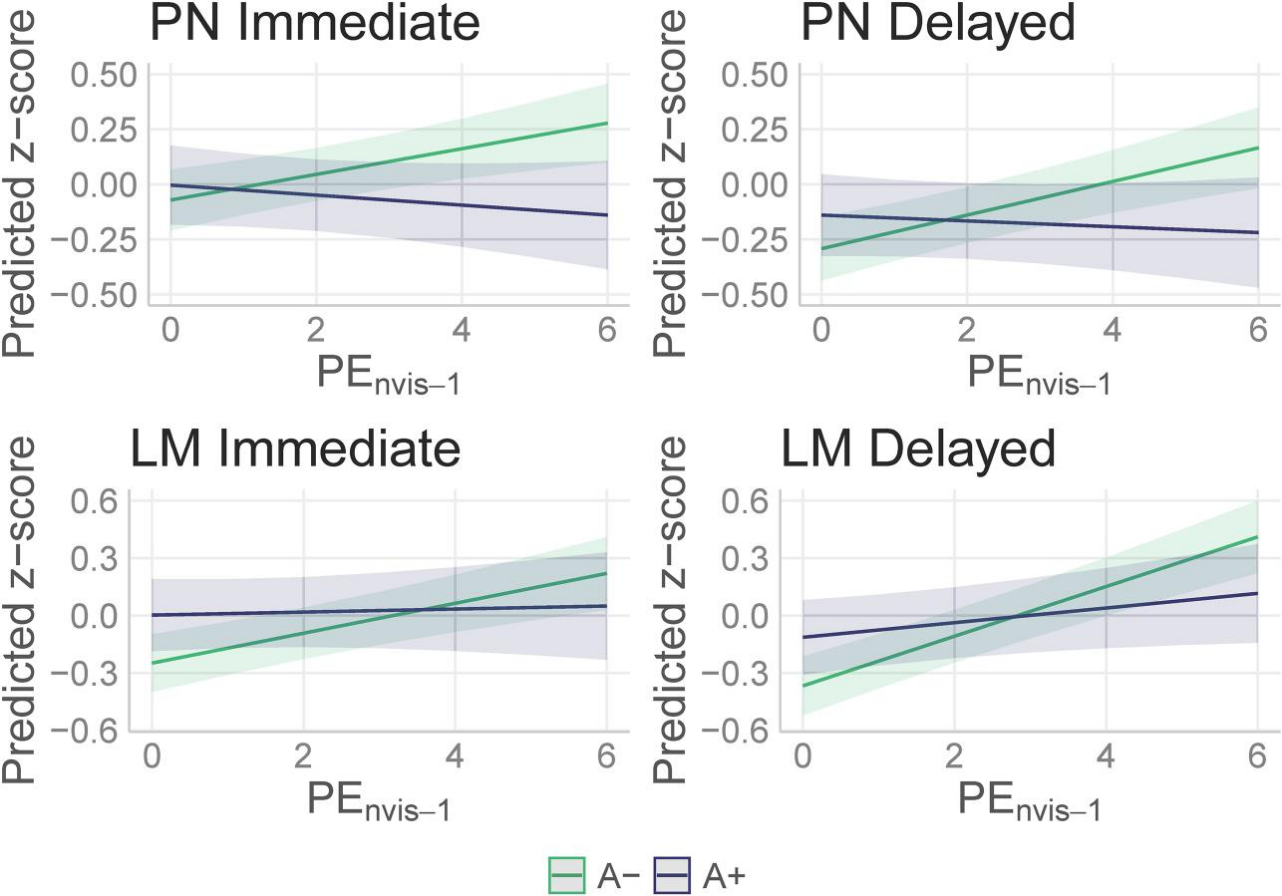
**Aim 2 model interactions: proper names and Logical Memory total score.** Interaction plots from linear mixed effects models illustrating the interaction between PE * amyloid status (reference group = A−; *P* < 0.001 for all outcomes) presented in Table 4 (*n* = 442). PN, proper name recall; LM, Logical Memory total score; PE_nvis-1_, Practice Effects (nvis-1 operationalization). A+, elevated amyloid PET (*n* = 116), A−, non-elevated amyloid PET (*n* = 326).

In our Aim 2 models, the proper names subscores showed a significant PE * amyloid status interaction (Table 4. Immediate: *β* = −0.08, *P* < 0.001, Delayed: *β* = −0.09, *P* < 0.001). The A+ group did not appear to benefit from practice as the A− group did [Fig. 2; simple slopes: Immediate: *β*_A−_ = 0.058 (0.024, 0.092), *β*_A+_ = −0.023 (−0.066, 0.021), Delayed: *β*_A−_ = 0.076 (0.043, 0.110), *β*_A+_ = −0.0132 (−0.056, 0.030)]. Effect sizes of the interaction terms in these models were very small-small (*η*^2^_PN Immediate_ = 0.008, *η*^2^_PN Delayed_ = 0.01).

**Table 4:** Aim 2: logical memory models

	PN—Immediate			PN—Delayed			LM—Immediate			LM—Delayed		
Predictors	*β*	CI	*P*	*β*	CI	*P*	*β*	CI	*P*	*β*	CI	*P*
(Intercept)	−3.49	−4.27 to −2.71	**<0**.**001**	−3.61	−4.43 to −2.79	**<0**.**001**	−4.26	−5.11 to −3.40	**<0**.**001**	−4.40	−5.28 to −3.53	**<0**.**001**
Age (centred)	−0.04	−0.05 to −0.03	**<0**.**001**	−0.04	−0.05 to −0.03	**<0**.**001**	−0.04	−0.05 to −0.03	**<0**.**001**	−0.05	−0.06 to −0.04	**<0**.**001**
Sex[female]	−0.04	−0.18 to 0.10	0.569	0.19	0.05 to 0.34	**0**.**010**	0.14	−0.01 to 0.29	0.076	0.19	0.03–0.34	**0**.**021**
WRAT-III	0.03	0.02 to 0.04	**<0**.**001**	0.03	0.02 to 0.04	**<0**.**001**	0.04	0.03–0.05	**<0**.**001**	0.04	0.03–0.05	**<0**.**001**
Age (centred)^2							−0.00	−0.00 to −0.00	**<0**.**001**	−0.00	−0.00 to −0.00	**<0**.**001**
PE (nvis-1)	0.06	0.02 to 0.09	**0**.**001**	0.08	0.04 to 0.11	**<0**.**001**	0.08	0.04 to 0.11	**<0**.**001**	0.13	0.10 to 0.16	**<0**.**001**
Amyloid status [+]	0.07	−0.10 to 0.24	0.441	0.15	−0.02 to 0.33	0.090	0.25	0.08 to 0.43	**0**.**005**	0.25	0.07 to 0.44	**0**.**006**
PE × Amyloid status [+]	−0.08	−0.12 to −0.04	**<0**.**001**	−0.09	−0.13 to −0.05	**<0**.**001**	−0.07	−0.11 to −0.03	**0**.**001**	−0.09	−0.13 to −0.06	**<0**.**001**
*N*	442 _WRAPNo_			442 _WRAPNo_			442 _WRAPNo_			442 _WRAPNo_		
Observations	2162			2163			2194			2194		
Marginal *R*^2^/Conditional *R*^2^	0.148/0.511			0.142/0.571			0.159/0.709			0.177/0.702		

Linear mixed effects models of logical memory and proper names subscores. PN, Proper Names; LM, Logical Memory (total score); PE, ractice Effects (nvis-1 operationalization utilized). Age was centred at 63.13 (SD = 7.70). Bolded *P*-values meeting significance threshold of *P* < 0.05.

In the sensitivity analyses of adding the PE * age interaction to our models, the PE * amyloid status interaction was no longer significant while the PE * age interaction was (Supplementary Table 2. Immediate: *β* = −0.03, *P* = 0.006, Delayed: *β* = −0.04, *P* = 0.003, Supplementary Fig. 2). In attempt to further tease apart the findings of the sensitivity analysis, we ran an additional set of models separating age into participants’ age at baseline and their time from baseline to potentially glean if it is overall age or decline over time that is impacting the A+ participants. However, these models had high multicollinearity and therefore the effects are difficult to disentangle and were not considered further for this paper.

### Aim 2: PExAmyloid Status—Logical Memory total score

In our Aim 2 analyses considering the interaction between PExA status, the Logical Memory scores showed a significant PE * amyloid status interaction (Table 4. Immediate: *β* = −0.07, *P* = 0.001, Delayed: *β* = −0.09, *P* < 0.001), such that those who were A+ did not improve as much with practice [Fig. 2; simple slopes: Immediate: *β*_PE|A+_ = 0.008 (−0.038, 0.054), *β*_PE|A−_ = 0.0781 (0.044, 0.112), Delayed: *β*_PE|A+_ = 0.038 (−0.002, 0.079), *β*_PE|A−_ = 0.130 (0.097, 0.163)]. Effect sizes of the interaction terms in these models were very small (*η*^2^_LM Immediate_ = 0.005, *η*^2^_LM Delayed_ = 0.007).

In our sensitivity analysis adding the age * amyloid status interaction to our Aim 2 models resulted in a non-significant PE * amyloid interaction (Supplementary Table 2, Immediate: *β* = 0.07, *P* = 0.10, Delayed: *β* = 0.06, *P* = 0.15) and a significant age * amyloid interaction (*β* = −0.06, *P* < 0.001, for both Immediate and Delayed outcomes). The sensitivity models’ simple slopes indicate that A+ participants are benefiting from practice [Immediate: *β* = 0.117 (0.047, 0.187), Delayed: *β* = 0.158 (0.089, 0.226)] comparably to A− participants [Immediate: *β* = 0.051 (0.015, 0.087), Delayed: *β* = 0.101 (0.065, 0.136)]. However, A+ participants are declining in Logical Memory outcomes more with age compared to the A− participants [Supplementary Fig. 1; Immediate: *β*_age|A+_ = −0.087 (−0.113, −0.062), *β*_age|A−_ = −0.029 (−0.042, −0.016), Delayed: *β*_age|A+_ = −0.093 (−0.117, −0.069), *β*_age|A−_ = −0.035 (−0.047, −0.022)].

### Aim 2: PExAmyloid Status—fluency outcomes

We explored the addition of amyloid status * PE_nvis-1_ to our base models. Animal Naming and CFL did not have a significant interaction between amyloid * PE (Supplementary Table 3; Animal Naming *β* = −0.01, *P* = 0.59, CFL *β* = −0.02, *P* = 0.19), but after removing the amyloid * PE interaction there was a significant main effect of PE such that with more practice, scores improved (Table 5; Animal Naming: *β* = 0.07, *P* < 0.001, CFL: *β* = 0.11, *P* < 0.001).

**Table 5:** Aim 2 fluency models

	Animal Naming			Letter fluency (CFL)		
Predictors	*β*	CI	*P*	*β*	CI	*P*
(Intercept)	−2.41	−3.29 to −1.52	**<0**.**001**	−3.66	−4.58 to −2.74	**<0**.**001**
Age (centred)	−0.04	−0.05 to −0.03	**<0**.**001**	−0.03	−0.04 to −0.02	**<0**.**001**
Sex [female]	−0.27	−0.43 to −0.11	**0**.**001**	−0.04	−0.20 to 0.13	0.672
WRAT-III reading score	0.02	0.02–0.03	**<0**.**001**	0.03	0.02–0.04	**<0**.**001**
PE (nvis-1)	0.07	0.03–0.10	**0**.**001**	0.11	0.08–0.15	**<0**.**001**
Amyloid status [+]	0.15	−0.02 to 0.32	0.093	0.17	−0.00 to 0.35	0.056
Age (centred)^2				−0.00	−0.00 to −0.00	**<0**.**001**
*N*	441 _WRAPNo_			442 _WRAPNo_		
Observations	1710			2191		
Marginal *R*^2^/Conditional *R*^2^	0.109/0.633			0.112/0.751		

Linear mixed effects models of fluency scores. PE, Practice Effects (nvis-1 operationalization utilized). Age was centred at 63.13 (SD = 7.70). Bolded *P*-values meeting significance threshold of *P* < 0.05.

### Aim 3: PExA/T Status—proper name recall

**Figure 3:**
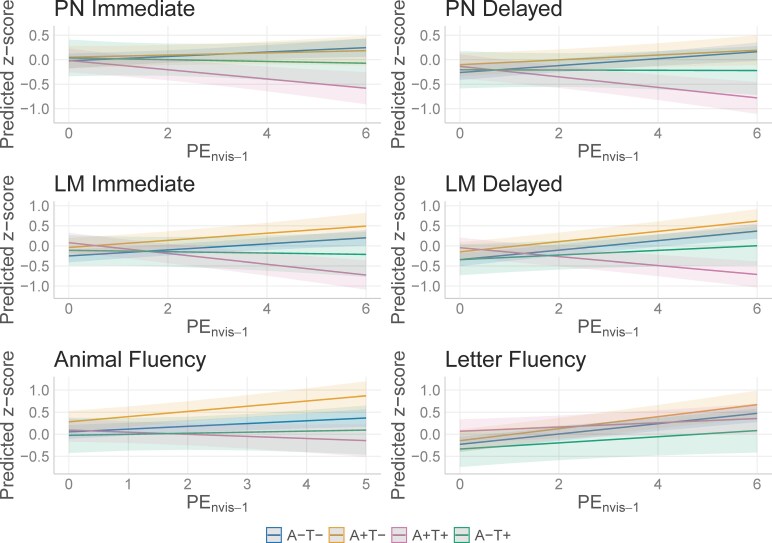
**Aim 3 model interactions.** Interaction plots from linear mixed effects models illustrating the interaction between PE * amyloid/tau status presented in Table 6 (PN & LM models, *n* = 397) and Table 7 (CFL and Animal Naming models, *n* = 396). PN, proper name recall, LM, Logical Memory total score, PE_nvis-1_, Practice Effects (nvis-1 operationalization). A+, elevated amyloid PET, A−, non-elevated amyloid PET, T+, elevated tau PET, T−, non-elevated tau PET. A−T− (*n* = 266) served as the reference group. Non-significant *P*-values were observed for the A+T− groups (*n* = 57, *P*’s 0.10–0.69). Significant *P*-values were observed for the LM Immediate A−T+ group (*n* = 20, *P* = 0.04; other outcomes *P*’s 0.10–0.51) and for all models A+T+ group (*n* = 54, *P*’s < 0.01).

In the Aim 3 analyses considering PExA/T status, we see a significant interaction between PE * A+T+ (Table 6. Immediate: *β* = −0.14, *P* < 0.001, Delayed: *β* = −0.18, *P* < 0.001), such that the A+T+ group is declining with practice [Fig. 3; simple slopes: Immediate: *β* = −0.094 (−0.154, −0.035), Delayed: *β* = −0.107 (−0.164, −0.051)] compared to the A−T− group (simple slopes: Immediate: *β* = 0.045 (0.009, 0.081), Delayed: *β* = 0.071 (0.035, 0.106)]. The effect size of the interaction terms in these models were small (*η*^2^_PN Immediate_ = 0.01, *η*^2^_PN Delayed_ = 0.03).

**Table 6:** Aim 3: Logical Memory and proper names models

	PN—Immediate			PN—Delayed			LM—Immediate			LM—Delayed		
Predictors	*β*	CI	*P*	*β*	CI	*P*	*β*	CI	*P*	*β*	CI	*P*
(Intercept)	−3.45	−4.26 to −2.64	**<0**.**001**	−3.65	−4.50 to −2.80	**<0**.**001**	−4.17	−5.05 to −3.30	**<0**.**001**	−4.31	−5.20 to −3.42	**<0**.**001**
Age (centred)	−0.04	−0.05 to −0.03	**<0**.**001**	−0.04	−0.05 to −0.03	**<0**.**001**	−0.04	−0.05 to −0.02	**<0**.**001**	−0.04	−0.05 to −0.03	**<0**.**001**
Sex [Female]	−0.05	−0.19 to 0.10	0.542	0.20	0.05 to 0.36	**0**.**009**	0.16	0.01 to 0.32	**0**.**042**	0.22	0.06 to 0.38	**0**.**007**
WRAT-III reading score	0.03	0.02 to 0.04	**<0**.**001**	0.03	0.02 to 0.04	**<0**.**001**	0.04	0.03 to 0.04	**<0**.**001**	0.04	0.03 to 0.05	**<0**.**001**
Age (centered)^2							−0.00	−0.00 to −0.00	**0**.**004**	−0.00	−0.00 to −0.00	**<0**.**001**
PE (nvis-1)	0.05	0.01 to 0.08	**0**.**015**	0.07	0.03 to 0.11	**<0**.**001**	0.08	0.04 to 0.11	**<0**.**001**	0.12	0.08 to 0.15	**<0**.**001**
A/T status [A+T−]	0.07	−0.16 to 0.30	0.530	0.16	−0.07 to 0.39	0.180	0.21	−0.02 to 0.44	0.071	0.19	−0.04 to 0.43	0.106
A/T status [A−T+]	0.06	−0.31 to 0.43	0.751	0.06	−0.32 to 0.43	0.772	0.14	−0.23 to 0.51	0.458	−0.00	−0.38 to 0.38	1.000
A/T status [A+T+]	0.01	−0.23 to 0.24	0.949	0.13	−0.12 to 0.37	0.308	0.33	0.09 to 0.57	**0**.**006**	0.30	0.06 to 0.54	**0**.**016**
PE × A/T status [A+T−]	−0.02	−0.08 to 0.03	0.423	−0.02	−0.07 to 0.03	0.407	0.01	−0.04 to 0.06	0.609	0.01	−0.03 to 0.05	0.686
PE × A/T status [A−T + ]	−0.06	−0.16 to 0.03	0.186	−0.07	−0.16 to 0.01	0.095	−0.09	−0.18 to −0.01	**0**.**036**	−0.06	−0.13 to 0.01	0.097
PE × A/T status [A+T+]	−0.14	−0.20 to −0.08	**<0**.**001**	−0.18	−0.23 to −0.12	**<0**.**001**	−0.21	−0.26 to −0.15	**<0**.**001**	−0.23	−0.27 to −0.18	**<0**.**001**
*N*	397 _WRAPNo_			397 _WRAPNo_			397 _WRAPNo_			397 _WRAPNo_		
Observations	1970			1971			2001			2001		
Marginal *R*^2^/Conditional *R*^2^	0.155/0.514			0.156/0.583			0.174/0.699			0.196/0.706		

Linear mixed effects models of Logical Memory total scores and Proper Name recall. PN, Proper name recall; LM, Logical Memory (total score); PE, Practice Effects (nvis-1 operationalization utilized); A/T, Amyloid/Tau status (+ = elevated, − = non-elevated). A−T− was the reference group for A/T status. Age was centred at 63.13 (SD = 7.70). Bolded *P*-values meeting significance threshold of *P* < 0.05.

In our sensitivity analysis, in which we added the PE * age interaction to the immediate recall model, the PE * A/T status was no longer significant for any group while the age * A/T status was significant for the A+T+ group (Supplementary Table 4. *β* = −0.04, *P* = 0.034). While all groups declined with time, the A+T+ group declined more per unit increase of time compared to the A−T− group [simple slopes: *β*_age|A−T−_ = −0.031 (−0.043, −0.019), *β*_age|A+T+_ = −0.069 (−0.102, −0.036)]. For the delayed recall model, the sensitivity analysis did not have any significant interactions with A/T status for either age or PE.

### Aim 3: PExA/T status—Logical Memory total score

In our Aim 3 analyses looking at PExA/T status, we observed a significant interaction between PE * A/T status for the A+T+ group on the Immediate Recall (Table 6. *β* = −0.21, *P* < 0.001) and the A−T+ group (*β* = −0.09, *P* = 0.036), such that these groups did not benefit from practice compared to the A−T− group [Fig. 3; simple slopes: *β*_PE|A+T+_ = −0.134 (−0.193, −0.075), *β*_PE|A−T+_ = −0.017 (−0.106, 0.072), *β*_PE|A−T−_ = 0.076 (0.040, 0.111)]. In our sensitivity analyses adding age * A/T status, significant interactions remained for PE in the A−T+ group for Immediate Recall (Supplementary Table 4. *β* = −0.22, *P* = 0.01) and were also observed for the A+T− group (*β* = 0.15, *P* = 0.005), but were no longer significant for the A+T+ group (*β*_PE|A+T+_ = −0.08, *P* = 0.12). There was also a significant interaction between age * A/T status for the A+T+ and A+T− groups (*β*_age|A+T+_ = −0.05, *P* = 0.008, *β*_age|A+T−_ = −0.06, *P* = 0.003), such that the A+T− group improved more with practice [simple slope: *β* = 0.200 (0.104, 0.295)] and the A−T+ group decreased more with practice [simple slope: *β* = −0.165, (−0.326, −0.003)] compared to the A−T− group [simple slope: *β* = 0.054 (0.015, 0.092)]. As for time, the A+T+ group and the A+T− group appear to be decreasing more over time [simple slopes: *β*_age|A+T+_ = −0.077 (−0.112, −0.041), *β*_age|A+T_− = −0.082 (−0.117, −0.047)] compared to the A−T− group [simple slope: *β* = −0.026 (−0.039, −0.012)].

In the Logical Memory—Delayed recall task, we saw a significant interaction between PE * A/T status for only the A+T+ group (Table 6. *β* = −0.23, *P* < 0.001) compared to the A−T− group, such that the A+T+ group has decreasing scores with each increase of practice [Fig. 3; simple slopes: *β*_PE|A+T+_ = −0.111 (−0.162, −0.060), *β*_PE*A−T−_ = 0.119 (0.085, 0.153)]. The effect sizes of these interaction terms were medium-large (*η*^2^_LM Immediate_ = 0.11, *η*^2^_LM Delayed_ = 0.06). In our sensitivity analysis including the age * A/T status interaction, the PE * A/T status was significant for all A/T groups compared to the A−T− group (Supplementary Table 4. A+T−: *β* = 0.17, *P* = 0.001, A−T+: *β* = −0.17, *P* = 0.037, A+T+: *β* = −0.13, *P* = 0.02). The simple slope estimates for each group showed that the A−T− and A+T− groups benefitted from practice [β_PE|A−T−_ = 0.096 (0.058, 0.133), *β*_PE|A+T−_ = 0.261 (0.169, 0.353)] while the A−T+ and A+T+ groups did not solidly improve or decline with practice [β_PE|A−T+_ = −0.075 (−0.231, 0.081), *β*_PE|A+T+_ = −0.031 (−0.131, 0.070)]. The age * A/T interaction term was significant for the A+T− (*β* = −0.06, *P* = 0.001) and A+T+ groups (*β* = −0.04, *P* = 0.03), such that these A/T groups decreased more than the A−T− group with time [simple slopes: *β*_age|A−T−_ = −0.029 (−0.042, −0.016), β_age|A+T−_ = −0.092 (−0.126, −0.058), *β*_age|A+T+_ = −0.069 (−0.104, −0.035)].

### Aim 3: PExA/T Status—fluency outcomes

We explored the interaction of A/T status * PE_nvis-1_ on our fluency outcomes. Both Animal Naming and CFL showed a significant A/T status * PE interaction (Table 7. Animal Naming: *β*_PE|A+T+_ = −0.11, *P* = 0.004, CFL: *β*_PE|A+T+_ = −0.07, *P* = 0.001), such that participants that were A+T+ did not benefit as much from practice compared to A−T− participants [Fig. 3; simple slopes: Animal Naming: *β*_PE|A+T+_ = 0.048 (0.122, 0.027), *β*_PE|A−T−_ = 0.063 (0.021, 0.105), CFL: *β*_PE|A_  _+_  _T+_=0.048 (−0.001, 0.098), *β*_PE|A-T−_ = 0.117 (0.082, 0.152). The effect sizes of these interaction terms were small (*η*^2^_Animal Naming_ = 0.01, *η*^2^_CFL_ = 0.009).

**Table 7:** Aim 3 fluency models

	Animal Naming			Letter fluency (CFL)		
Predictors	*β*	CI	*P*	*β*	CI	*P*
(Intercept)	−2.48	−3.38 to −1.58	**<0**.**001**	−3.73	−4.70 to −2.76	**<0**.**001**
Age (centred)	−0.04	−0.05 to −0.03	**<0**.**001**	−0.03	−0.04 to −0.01	**<0**.**001**
Sex [female]	−0.24	−0.40 to −0.08	**0**.**004**	0.00	−0.17 to 0.18	0.981
WRAT-III reading score	0.02	0.02 to 0.03	**<0**.**001**	0.03	0.02 to 0.04	**<0**.**001**
Age (centred)^2				−0.00	−0.00 to −0.00	0.003
PE (nvis-1)	0.06	0.02 to 0.11	**0**.**003**	0.12	0.08 to 0.15	**<0**.**001**
AT bin [A+T−]	0.23	−0.02 to 0.47	0.068	0.08	−0.17 to 0.33	0.521
AT bin [A−T+]	−0.08	−0.47 to 0.32	0.696	−0.11	−0.51 to 0.30	0.605
AT bin [A+T+]	0.04	−0.22 to 0.30	0.756	0.30	0.04 to 0.56	**0**.**025**
PE × AT bin [A+T−]	0.05	−0.01 to 0.12	0.101	0.02	−0.02 to 0.06	0.336
PE × AT bin [A−T+]	−0.04	−0.15 to 0.08	0.507	−0.05	−0.11 to 0.02	0.161
PE × AT bin [A+T+]	−0.11	−0.18 to −0.04	**0**.**004**	−0.07	−0.11 to −0.03	**0**.**001**
*N*	396 _WRAPNo_			397 _WRAPNo_		
Observations	1567			1998		
Marginal *R*^2^/Conditional *R*^2^	0.131/0.637			0.117/0.750		

Linear mixed effects models of fluency outcomes. PE, Practice Effects (nvis-1 operationalization utilized); A/T, Amyloid/Tau status (+ = elevated, − = non-elevated). A−T− was the reference group for A/T status. Age was cantered at 63.13 (SD = 7.70). Bolded *P*-values meeting significance threshold of *P* < 0.05.

Our sensitivity analyses of adding the interaction of age * A/T status showed that the interaction of A/T status * PE was no longer significant comparing the A+T+ group to the A−T− reference group, but for the Animal Naming task this A/T group comparison was significant for the age * A/T status group variable (Supplementary Table 5, *β*_age|A+T+_ = −0.05, *P* = 0.01), such that participants that were A+T+ had lower scores over time [simple slopes: *β*_age|A+T+_ = −0.078 (−0.112, −0.044, *β*_age|A−T−_ = −0.0305 (−0.044, −0.017)]. In the CFL, the sensitivity analyses adding in the age * A/T status had significant interactions of PE * A+T− (Supplementary Table 5. *β*_PE|A+T−_ = 0.17, *P* = 0.001) such that additional practice increased CFL score compared to the A−T− group (simple slopes: *β*_PE|A+T−_ = 0.273 (0.179, 0.367), *β*_PExA−T−_ = 0.102 (0.063, 0.141)] and age * A+T− (*β*_age|A+T−_ = −0.06, *P* = 0.002) such that CFL scores decreased more over time compared to the A−T− group [simple slopes *β*_age|A+T−_ = −0.080 (−0.115, −0.045), *β*_age|A−T−_ = −0.019 (−0.033, −0.005)].

## Discussion

In this study we used linear mixed effects models to demonstrate a novel relationship between PEs, language-based neuropsychological measures (proper name recall from Logical Memory, Logical Memory total scores, letter fluency and category fluency), and PET biomarkers of AD. Linear mixed effects models investigating three different operationalizations found that our operationalization of PE_nvis-1_ generally performed the best (Aim 1). We then looked at the interactions between PE, cognitive tests and amyloid and tau biomarker positivity (Aims 2 and 3) using linear mixed effects models. We found that amyloid may moderate PE, such that individuals with elevated amyloid did not benefit as much from practice on proper name recall and Logical Memory total score compared to participants who were amyloid negative. When considering the additional impact of tau and its interactions in our models, we found that for proper name recall and Logical Memory total score, amyloid and tau statuses both may moderate the impact of PE, such that those who were positive for both biomarkers declined on proper name recall and Logical Memory total scores compared to those who were biomarker negative. However, when we considered a sensitivity analysis of introducing an interaction of age * biomarker status to our models, the moderating impact that we previously saw with PE mostly disappeared, indicating that perhaps age or time was contributing more to the impact we were observing. This highlights the need to attempt to further disentangle age, time and PE in future studies, although other research suggests these items may not be able to be distinguished from one another.^35^

Our Aim 1 results testing different proposed operationalizations of PE—specifically, number of cognitive task exposures − 1 (‘PE_nvis-1_’), the square root of PE_nvis-1_ (‘PE_sqrt_’) and a categorical measure coding numbers of exposures as 0, 1, 2 or 3 or more (‘PE_cat_’)—indicate that the measures we utilized performed similarly, with a difference of AIC that was less than 5 points between the operationalizations in nearly all models, with an exception of the CFL model, which indicated that the PE_cat_ operationalization provided less utility, and the Logical Memory delayed total score, which had the lowest AIC for the PE_cat_ operationalization The PE_nvis-1_ was likely more often sufficient due to the additional power provided by the nature of being a continuous variable. However, in some circumstances the categorical operationalization (PE_cat_) may be useful in comparing how the different amounts of practice (1, 2 and 3 or more exposures to the task) relate in comparison to the baseline visit. Other researchers have performed similar techniques to ours, utilizing linear mixed effects models to assess PE.^20,38^ In addition to using linear mixed effects models, categorical operationalizations have been used.^40^ Other studies in PE look at short-term changes utilizing change scores.^19,41^ Accounting for PE using any of these measures would be good practice in repeated measures designs and longitudinal studies.

In Aim 2, amyloid positive participants showed a decline in scores on proper name recall and Logical Memory total score with more practice for both Immediate and Delayed Recall. These results suggest that both learning (immediate recall) and memory (delayed recall) declined with practice in the amyloid positive participants for both the overall Logical Memory task and the proper names subscores. Previous studies examining PE in the WRAP cohort failed to demonstrate PE for the Logical Memory story recall tasks.^24^ Our study includes more follow-up data that has accumulated since the previous study was published in 2015, which may impact the presence of PE we observed. The CFL and Animal Naming tasks did not produce a significant interaction for this aim and only demonstrated a main effect for practice. The association between amyloid positivity and decreased PE on Logical Memory outcomes is similar to previous findings by Duff and colleagues who used the BVMT-R assessing visual learning.^17^ These findings were expanded upon by Duff and colleagues to more tests including the Hopkins Verbal Learning Test, Symbol Digit Modalities and Trails A and B to find similar results, indicating that PE may be indicative of AD pathology, with an odds ratio of being amyloid positive found to be 13.5 times higher in participants with low, short-term PE.^41^ Our findings in Logical Memory and Duff and colleagues’ findings demonstrate that both learning and memory are subjected to moderation of PE by amyloid biomarkers. However, in our case, sensitivity analyses indicated that it may perhaps be age that is driving this difference as our amyloid positive group (average baseline age = 59.68) is generally older than our amyloid negative group (average baseline age = 57.15, *t* = −4.02, *P* < 0.001).

Our findings that amyloid moderates PE in proper names and Logical Memory total score indicate that these scores may be more sensitive to PE and amyloid than other measures. This possible increased sensitivity could be explained by lesion and functional magnetic resonance imaging studies demonstrating that proper name recall is mediated by the left and right anterior temporal lobe and the parahippocampal gyri, which are also sites of early neuropathology accumulation in AD.^11,12,42,43^ This overlap in areas of mediation and biomarker build up serves as a biological basis for why those individuals with amyloid build up do not benefit from continued practice on the same material as those without amyloid positivity. Although this finding requires replication and additional investigation, it provides information to AD researchers on possible cognitive tasks where PE will possibly not skew results.

The finding that CFL and Animal Naming tasks had a main effect of PE has been seen before within the WRAP cohort,^44^ though it was not explicitly explored and was only included as a covariate. While the neural correlates of CFL and Animal Naming tasks are areas involved in early AD biomarker accumulation, such as the temporal-parietal region, inferior frontal gyrus and anterior cingulate^45,46^ verbal fluency tasks also involve regions not involved with early amyloid and tau accumulation, like the cerebellum and thalamus. This additional activation is likely because these tasks are mediated by additional factors such as inhibition, information monitoring, semantics, phonological assembly and age of acquisition.^47^ It is possible that the relatively young age and cognitively unimpaired status of the current cohort means that we were not yet able to detect an association with these tasks and amyloid positivity, which was seen in other, more cognitively impaired populations.^48^ However, as the cohort ages and more participants may become impaired, this association might become detectable.

In Aim 3, we investigated whether tau positivity explained additional variance beyond amyloid positivity in relation to PE. Once we included combined A/T status in the analyses, the interaction of A/TxPE was significantly associated with Logical Memory and Proper Names subscores for both Immediate and Delayed tasks and for CFL and Animal Naming, such that A+T+ participants had a decrease in score with each increased unit of practice compared to the A−T− participants. Our sensitivity analyses, adding in the PExA/T status + agexA/T status showed some interesting results. We saw that for A+T− participants, there were significant *P*-values with positive beta values for the Animal Naming, CFL, and Logical Memory Immediate and Delayed outcomes, indicating that A+T− participants were benefitting more from practice compared to A-T- individuals. We hypothesize that these participants in the early stages of the AD continuum may be experiencing cognitive reserve,^49^ which has been shown in previous studies to be associated with improved performance in unimpaired, A+ participants.^50^

The inclusion of tau in our analyses indicates that participants further along the AD continuum may benefit less from practice. Young and colleagues^51^ have also looked at the impact of tau on PE and found that within short-term practice schedules, PE is sensitive to tau burden more so than amyloid. While the majority of studies considering PE seem to use short-term practice, it is important to be able to understand the impacts of PE in longitudinal studies, since these methods may be closer to what would be experienced in a timeline in a clinical setting, which is an important addition that our work is adding to the literature.

Other studies have considered the impact of neurodegeneration, which found that neurodegeneration had a stronger influence on practice than amyloid status.^52^ While we did not consider the impact of neurodegeneration, it might prove useful to include ATN status in future analyses to determine if one biomarker produces more of an impact on practice and may be driving the impact of practice on disease progression. In a more general sense, an absence of PE have been found to be a potential marker of AD, even in unimpaired populations,^53^ similar to the WRAP cohort. An absence of PE in unimpaired participants has been shown to be predictive of autopsy confirmed AD,^20^ which indicates that PE may be another sensitive measure of preclinical AD.^54^ Given the nature of AD clinical trials targeting the preclinical phase of the disease,^55^ and the expenses associated with genetic testing and PET imaging,^56^ use of PE in sensitive tests like Logical Memory may be a cost effective way to consider who to include in clinical trials or for whom to pursue further confirmatory testing.

Our study has several limitations that should be considered. One limitation is the generalizability of the WRAP cohort, as the majority of participants are well-educated and identify as non-Hispanic white, meaning these findings may not generalize to the population as a whole or the population of those with AD. Further compounding this limitation, the racial makeup of the participants who have received PET scans also consists of mostly white, highly educated participants. Previous studies have found that Black/African Americans are less willing to enroll in AD research^57^ and specific themes related to discouraging biomarker testing, such as participant burden/harm, low personal utility, potential for stigma and discrimination, and potential psychological consequences have been explored.^58^ Due to efforts within the WRAP study to address potential barriers to study participation, future analyses will have a larger sample of participants from more diverse backgrounds with longitudinal cognitive data and amyloid PET scans. An additional limitation is that we did not run a priori power calculations due to the fact that proper names from story recall have not been investigated with PE. Therefore, it is possible that our study was underpowered to detect associations between proper name recall and PE. Upon testing our models for partial eta square effect sizes, we found that we had mostly very small to small effect sizes, with the exception of a medium effect size for the Logical Memory tasks, when considering our PExbiomarker status interaction terms (range: < 0.001–0.11), which indicates we may have been underpowered to appropriately detect differences. While our overall sample size was large (*n* = 442), we had unequal group sizes for our biomarkers of interest. In addition, our cohort is mostly healthy and biomarker negative (∼74% A−; ∼67% A−/T−), which may have limited power in our models. Lastly, the use of binary variables to represent biomarker status means that some participants with slightly lower accumulation of amyloid or tau may have been missed as they did not reach the threshold for biomarker positivity. These limitations point the need to replicate the findings of the current study in additional and more diverse cohorts.

A significant strength of the current study is the use of a widely used neuropsychological test, Logical Memory, to further investigate the relationship between language-based measures and PE. Due to this fact, there is a large amount of already existing data that could be used to replicate the findings of the current study. Second, this study investigated the association between PE and A/T status. This inclusion provides a fuller picture of the interaction between PE and biomarkers of AD in this preclinical/early stage of the disease. Lastly, this study tested three differing operationalizations of PE prior to implementing our models necessitating a variable quantifying PE, and that a simple metric—the total number of exposures to the test minus 1 (baseline)—was sufficient to capture practice among most measures. This allowed us to ensure we implemented the most appropriate operationalization for each measure of interest.

PE are often treated as a nuisance, either through study design seeking to mitigate PE or through controlling for such effects in statistical modelling^59^; however, from the current study we see that PE can be an indicator of biomarker positivity that may indicate future cognitive decline. Due to the time, expenses and burden to participants in undergoing PET scans, considering PE as an indicator of possible progression to AD can diminish the need for undergoing such procedures. This knowledge can be leveraged to provide us with more detail to as to who may progress along the AD continuum before clinical manifestations occur.

Further investigation into teasing apart aging, time and PE is needed to better understand this complex, interconnected relationship. Additional future directions for this work include repeating with other biomarkers, such as plasma pTau-217 or markers of neurodegeneration. It could also be beneficial to update the method of determining participants’ tau status, as the preferred ROI has evolved in recent years. It would also be useful to re-run the analyses when we have a larger population of underrepresented groups. Additionally, it would be beneficial to test language measures and PE with other cohorts that might have more biomarker positive or cognitively impaired participants. We also plan to repeat these analyses exploring nonlinear methods. Lastly, previous work has used PE to predict later cognition, which could also be done in the WRAP cohort.

Our results suggest that PE may moderate the association between amyloid, tau and Logical Memory total score and proper names subscores. Specifically, we found that individuals with amyloid and tau positivity did not benefit from PE on these measures to the same extent that amyloid negative individuals did, with both amyloid and tau statuses moderating the effect of practice on Logical Memory Immediate and Delayed recall tasks. These findings suggest that these cognitive measures combined with measures of practice, may be particularly useful when investigating long-term changes in cognition in individuals at risk for AD. Validation of the results from the current study by replication in additional cohorts is needed.

## Supplementary Material

fcaf390_Supplementary_Data

## Data Availability

The datasets presented in this article are not readily available because data are available through a data request process. Requests to access the datasets should be directed to https://wrap.wisc.edu/data-requests/. Access will be granted to named individuals in accordance with ethical procedures governing the reuse of sensitive data. Additionally, requestors must meet the following condition to obtain access: completion of a formal data sharing agreement.
