# Supplementary material for: Amyloid and tau may moderate practice effects in semantic and episodic memory in a cognitively unimpaired at-risk sample
Source: Brain Commun. 2025 Oct 9;7(5):fcaf390. doi: 10.1093/braincomms/fcaf390 (PMC12569600; doi:10.1093/braincomms/fcaf390)
Supplement: fcaf390_Supplementary_Data [file fcaf390_supplementary_data.docx]

**Supplemental Table 1. Base Models of Practice**

|  | **Animal Naming** | | | **CFL** | | | **PN - Immediate** | | | **PN - Delayed** | | | **LM - Immediate** | | | **LM - Delayed** | | |
| --- | --- | --- | --- | --- | --- | --- | --- | --- | --- | --- | --- | --- | --- | --- | --- | --- | --- | --- |
| *Predictors* | *β* | *CI* | *p* | *β* | *CI* | *p* | *β* | *CI* | *p* | *β* | *CI* | *p* | *β* | *CI* | *p* | *β* | *CI* | *p* |
| (Intercept) | -2.36 | -3.25 –  -1.48 | **<0.001** | -3.61 | -4.53 –  -2.69 | **<0.001** | -3.48 | -4.26 –  -2.70 | **<0.001** | -3.58 | -4.40 –  -2.75 | **<0.001** | -4.19 | -5.05 –  -3.33 | **<0.001** | -4.33 | -5.21 –  -3.46 | **<0.001** |
| Age (centered) | -0.04 | -0.05 –  -0.03 | **<0.001** | -0.03 | -0.04 –  -0.01 | **<0.001** | -0.04 | -0.05 –  -0.03 | **<0.001** | -0.04 | -0.05 –   -0.03 | **<0.001** | -0.04 | -0.05 –  -0.03 | **<0.001** | -0.05 | -0.06 –  -0.04 | **<0.001** |
| Sex [female] | -0.26 | -0.42 –   -0.11 | **0.001** | -0.03 | -0.19 –  0.14 | 0.765 | -0.05 | -0.19 –   0.09 | 0.509 | 0.19 | 0.05 –   0.34 | **0.010** | 0.15 | -0.01 –  0.30 | 0.060 | 0.19 | 0.03 – 0.34 | **0.019** |
| WRAT-III | 0.02 | 0.02 –  0.03 | **<0.001** | 0.03 | 0.02 –   0.04 | **<0.001** | 0.03 | 0.02 –  0.04 | **<0.001** | 0.03 | 0.02 –  0.04 | **<0.001** | 0.04 | 0.03 – 0.05 | **<0.001** | 0.04 | 0.03 – 0.05 | **<0.001** |
| PE | 0.06 | 0.02 –   0.10 | **0.001** | 0.11 | 0.08 –  0.14 | **<0.001** | 0.04 | 0.01 –  0.07 | **0.018** | 0.05 | 0.02 –   0.08 | **0.001** | 0.06 | 0.02 – 0.09 | **0.001** | 0.10 | 0.07 – 0.14 | **<0.001** |
| Age (centered)^2 |  |  |  | -0.00 | -0.00 –   -0.00 | **<0.001** |  |  |  |  |  |  | -0.00 | -0.00 –  -0.00 | **<0.001** | -0.00 | -0.00 –  -0.00 | **<0.001** |
| **Random Effects** | | | | | | | | | | | | | | | | | | |
| σ^2^ | 0.36 | | | 0.24 | | | 0.50 | | | 0.44 | | | 0.30 | | | 0.31 | | |
| τ_00_ | 0.52 _WRAPNo_ | | | 0.62 _WRAPNo_ | | | 0.37 _WRAPNo_ | | | 0.43 _WRAPNo_ | | | 0.48 _WRAPNo_ | | | 0.54 _WRAPNo_ | | |
| τ_11_ |  | | |  | | |  | | |  | | | 0.00 _WRAPNo.c_curage_ | | |  | | |
| ρ_01_ |  | | |  | | |  | | |  | | | 0.22 _WRAPNo_ | | |  | | |
| ICC | 0.59 | | | 0.72 | | | 0.42 | | | 0.50 | | | 0.66 | | | 0.63 | | |
| N | 441 _WRAPNo_ | | | 442 _WRAPNo_ | | | 442 _WRAPNo_ | | | 442 _WRAPNo_ | | | 442 _WRAPNo_ | | | 442 _WRAPNo_ | | |
| Observations | 1710 | | | 2191 | | | 2162 | | | 2163 | | | 2194 | | | 2194 | | |
| Marginal R^2^ / Conditional R^2^ | 0.106 / 0.633 | | | 0.107 / 0.751 | | | 0.142 / 0.506 | | | 0.137 / 0.566 | | | 0.156 / 0.712 | | | 0.174 / 0.698 | | |

Linear-mixed effects models of neuropsychological tests without biomarkers present in models. PN=Proper Names, LM=Logical Memory (Total Score), PE=Practice Effects (nvis-1 operationalization utilized). Age was centered at 63.13 (sd=7.70). Bolded p-values meeting significance threshold of p<0.05.

**Supplemental Table 2. Aim 2 Sensitivity Analysis: Logical Memory and Proper Names Models with AgexAmloid Status**

|  | **PN – Immediate** | | | **PN – Delayed** | | | **LM – Total Score Immediate** | | | **LM – Total Score Delayed** | | |
| --- | --- | --- | --- | --- | --- | --- | --- | --- | --- | --- | --- | --- |
| *Predictors* | *β* | *CI* | *p* | *β* | *CI* | *p* | *β* | *CI* | *p* | *β* | *CI* | *p* |
| (Intercept) | -3.45 | -4.23 –   -2.68 | **<0.001** | -3.56 | -4.38 –   -2.75 | **<0.001** | -4.18 | -5.03 –   -3.33 | **<0.001** | -4.34 | -5.21 –   -3.46 | **<0.001** |
| Age (centered) | -0.03 | -0.05 –  -0.02 | **<0.001** | -0.03 | -0.04 –   -0.02 | **<0.001** | -0.03 | -0.04 –   -0.02 | **<0.001** | -0.03 | -0.05 –   -0.02 | **<0.001** |
| Gender [female] | -0.04 | -0.18 –   0.10 | 0.558 | 0.19 | 0.05 – 0.34 | **0.009** | 0.14 | -0.02 –   0.29 | 0.079 | 0.19 | 0.03 – 0.34 | **0.020** |
| WRAT-III | 0.03 | 0.02 – 0.04 | **<0.001** | 0.03 | 0.02 – 0.04 | **<0.001** | 0.04 | 0.03 –  0.05 | **<0.001** | 0.04 | 0.03 – 0.05 | **<0.001** |
| Age (centered)^2 |  |  |  |  |  |  | -0.00 | -0.00 –   -0.00 | **0.001** | -0.00 | -0.00 –  -0.00 | **<0.001** |
| PE (nvis-1) | 0.04 | 0.01 – 0.08 | **0.022** | 0.06 | 0.02 – 0.09 | **0.002** | 0.05 | 0.02 –  0.09 | **0.005** | 0.10 | 0.07 – 0.14 | **<0.001** |
| Amyloid status [+] | -0.08 | -0.27 –   0.12 | 0.458 | -0.01 | -0.21 –  0.20 | 0.930 | -0.00 | -0.21 –  0.21 | 0.999 | 0.02 | -0.19 –  0.23 | 0.869 |
| PE × Amyloid status [+] | 0.01 | -0.07 –  0.08 | 0.830 | 0.01 | -0.07 –   0.09 | 0.774 | 0.07 | -0.01 –   0.14 | 0.097 | 0.06 | -0.02 –  0.13 | 0.146 |
| Age (centered) × Amyloid status [+] | -0.03 | -0.06 –  -0.01 | **0.006** | -0.04 | -0.07 –  -0.01 | **0.003** | -0.06 | -0.09 –  -0.03 | **<0.001** | -0.06 | -0.09 –  -0.03 | **<0.001** |
| **Random Effects** | | | | | | | | | | | | |
| σ^2^ | 0.50 | | | 0.43 | | | 0.31 | | | 0.31 | | |
| τ_00_ | 0.36 _WRAPNo_ | | | 0.43 _WRAPNo_ | | | 0.48 _WRAPNo_ | | | 0.53 _WRAPNo_ | | |
| τ_11_ |  | | |  | | | 0.00 _WRAPNo.c_curage_ | | |  | | |
| ρ_01_ |  | | |  | | | 0.23 _WRAPNo_ | | |  | | |
| ICC | 0.42 | | | 0.50 | | | 0.65 | | | 0.64 | | |
| N | 442 _WRAPNo_ | | | 442 _WRAPNo_ | | | 442 _WRAPNo_ | | | 442 _WRAPNo_ | | |
| Observations | 2162 | | | 2163 | | | 2194 | | | 2194 | | |
| Marginal R^2^ / Conditional R^2^ | 0.154 / 0.511 | | | 0.150 / 0.571 | | | 0.175 / 0.710 | | | 0.193 / 0.706 | | |

Linear-mixed effects models of Logical Memory total scores and Proper Names subscores. PN=Proper Names, LM=Logical Memory (Total Score), PE=Practice Effects (nvis-1 operationalization utilized). Age was centered at 63.13 (sd=7.70). Bolded p-values meeting significance threshold of p<0.05.

**Supplemental Figure 1. Aim 2: Logical Memory and Proper Names Models with AgexAmyloid Status**


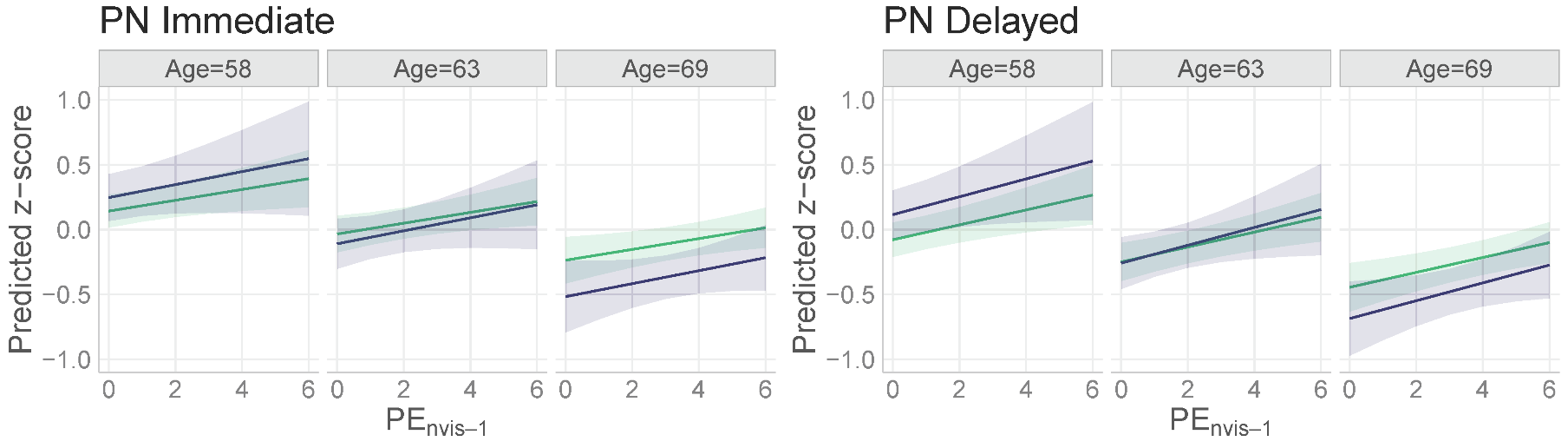


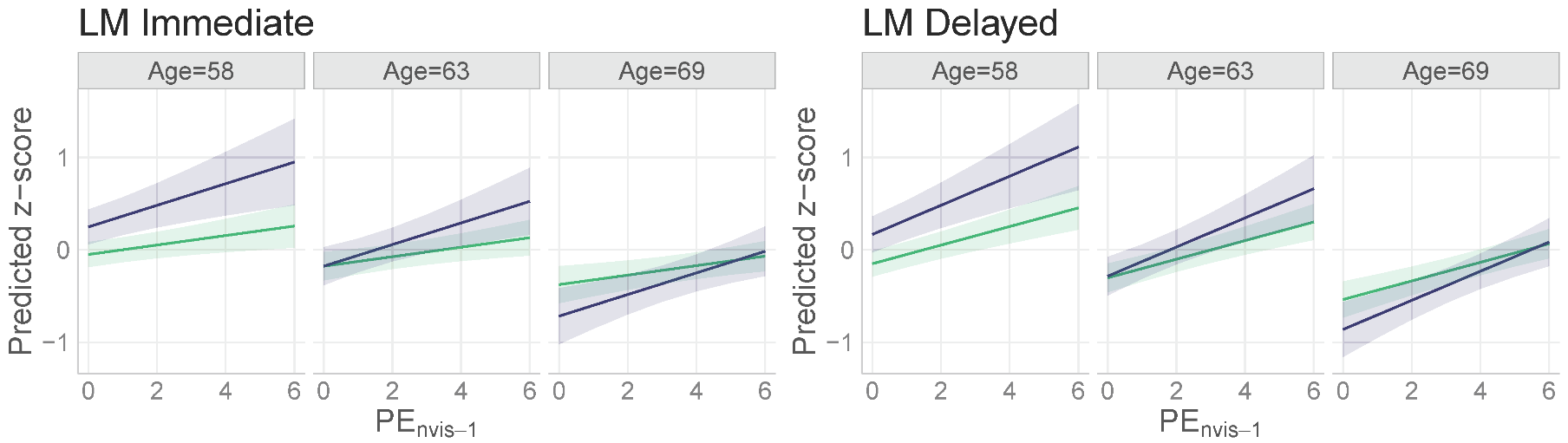


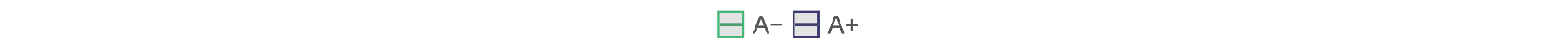


Interaction plots from linear mixed effects models presented in Supplemental Table1 (n=442), displaying the practice across different ages (1^st^ quartile=58, Mean age=63, 2^nd^ quartile=69). PN=Proper name recall, LM=Logical Memory total score, PE_nvis-1_=Practice Effects (nvis-1 operationalization). A+=elevated amyloid PET (n=116), A-=non-elevated amyloid PET (n=326).

**Supplemental Table 3. Aim 2: Fluency Models (with Amyloid*PE interaction)**

|  | **Animal Naming** | | | **CFL** | | |
| --- | --- | --- | --- | --- | --- | --- |
| *Predictors* | *β* | *CI* | *p* | *β* | *CI* | *p* |
| (Intercept) | -2.41 | -3.29 – -1.53 | **<0.001** | -3.67 | -4.59 – -2.75 | **<0.001** |
| Age (centered) | -0.04 | -0.05 – -0.03 | **<0.001** | -0.03 | -0.04 – -0.02 | **<0.001** |
| Gender [female] | -0.27 | -0.43 – -0.11 | **0.001** | -0.04 | -0.20 – 0.13 | 0.674 |
| WRAT-III | 0.02 | 0.02 – 0.03 | **<0.001** | 0.03 | 0.02 – 0.04 | **<0.001** |
| PE (nvis-1) | 0.07 | 0.03 – 0.11 | **0.001** | 0.12 | 0.09 – 0.15 | **<0.001** |
| Amyloid status [+] | 0.17 | -0.02 – 0.35 | 0.080 | 0.21 | 0.03 – 0.40 | **0.026** |
| PE × Amyloid status [+] | -0.01 | -0.07 – 0.04 | 0.593 | -0.02 | -0.05 – 0.01 | 0.192 |
| Age (centered)^2 |  |  |  | -0.00 | -0.00 – -0.00 | **0.001** |
| **Random Effects** | | | | | | |
| σ^2^ | 0.36 | | | 0.24 | | |
| τ_00_ | 0.52 _WRAPNo_ | | | 0.61 _WRAPNo_ | | |
| ICC | 0.59 | | | 0.72 | | |
| N | 441 _WRAPNo_ | | | 442 _WRAPNo_ | | |
| Observations | 1710 | | | 2191 | | |
| Marginal R^2^ / Conditional R^2^ | 0.109 / 0.633 | | | 0.112 / 0.751 | | |

Linear-mixed effects models of fluency measures. CFL=letter fluency (C,F,L), PE=Practice Effects (nvis-1 operationalization utilized). Age was centered at 63.13 (sd=7.70). Bolded p-values meeting significance threshold of p<0.05.

**Supplemental Table 4. Aim 3 Sensitivity Analysis: Logical Memory and Proper Name Models with AgexA/T Status**

|  | **PN – Immediate** | | | **PN – Delayed** | | | **LM –Immediate** | | | **LM – Delayed** | | |
| --- | --- | --- | --- | --- | --- | --- | --- | --- | --- | --- | --- | --- |
| *Predictors* | *β* | *CI* | *p* | *β* | *CI* | *p* | *β* | *CI* | *p* | *β* | *CI* | *p* |
| (Intercept) | -3.43 | -4.23 –  -2.62 | **<0.001** | -3.61 | -4.46 –  -2.77 | **<0.001** | -4.11 | -4.98 –  -3.24 | **<0.001** | -4.25 | -5.13 –  -3.38 | **<0.001** |
| Age (centered) | -0.03 | -0.04 –  -0.02 | **<0.001** | -0.03 | -0.04 –  -0.02 | **<0.001** | -0.03 | -0.04 –   -0.01 | **<0.001** | -0.03 | -0.04 –  -0.02 | **<0.001** |
| Gender [Female] | -0.05 | -0.19 –  0.09 | 0.499 | 0.20 | 0.05 – 0.35 | **0.010** | 0.16 | -0.00 –  0.31 | 0.051 | 0.21 | 0.06 – 0.37 | **0.008** |
| WRAT-III Reading Score | 0.03 | 0.02 – 0.04 | **<0.001** | 0.03 | 0.02 – 0.04 | **<0.001** | 0.04 | 0.03 – 0.04 | **<0.001** | 0.04 | 0.03 – 0.05 | **<0.001** |
| Age (centered)^2 |  |  |  |  |  |  | -0.00 | -0.00 –   -0.00 | **0.007** | -0.00 | -0.00 –  -0.00 | **<0.001** |
| PE (nvis-1) | 0.03 | -0.01 –  0.07 | 0.123 | 0.05 | 0.02 – 0.09 | **0.006** | 0.05 | 0.02 – 0.09 | **0.006** | 0.10 | 0.06 – 0.13 | **<0.001** |
| A/T status [A+T-] | -0.05 | -0.33 –   0.22 | 0.694 | 0.00 | -0.27 –  0.28 | 0.981 | -0.05 | -0.33 –  0.23 | 0.729 | -0.07 | -0.35 –  0.20 | 0.601 |
| A/T status [A-T+] | 0.07 | -0.31 –   0.45 | 0.718 | 0.05 | -0.34 –   0.43 | 0.817 | 0.21 | -0.19 –   0.60 | 0.302 | 0.02 | -0.37 –  0.41 | 0.929 |
| A/T status [A+T+] | -0.14 | -0.40 –  0.13 | 0.321 | -0.01 | -0.28 –   0.27 | 0.950 | 0.12 | -0.15 –  0.40 | 0.386 | 0.14 | -0.14 –   0.42 | 0.318 |
| PE × A/T status [A+T-] | 0.05 | -0.05 –  0.15 | 0.346 | 0.07 | -0.04 –   0.17 | 0.202 | 0.15 | 0.04 – 0.25 | **0.005** | 0.17 | 0.07 – 0.26 | **0.001** |
| PE × A/T status [A-T+] | -0.13 | -0.29 –  0.04 | 0.131 | -0.11 | -0.28 –  0.05 | 0.185 | -0.22 | -0.38 –   -0.05 | **0.010** | -0.17 | -0.33 –   -0.01 | **0.037** |
| PE × A/T status [A+T+] | -0.04 | -0.15 –  0.07 | 0.478 | -0.09 | -0.20 –  0.02 | 0.115 | -0.08 | -0.19 –  0.02 | 0.123 | -0.13 | -0.23 –  -0.02 | **0.020** |
| Age (centered) × A/T status [A+T-] | -0.03 | -0.06 –  0.01 | 0.101 | -0.04 | -0.07 –  0.00 | 0.051 | -0.06 | -0.09 –   -0.02 | **0.003** | -0.06 | -0.10 –   -0.03 | **0.001** |
| Age (centered) × A/T status [A-T+] | 0.03 | -0.03 –   0.08 | 0.357 | 0.01 | -0.04 –  0.07 | 0.598 | 0.05 | -0.01 –   0.11 | 0.093 | 0.04 | -0.01 –   0.10 | 0.145 |
| Age (centered) × A/T status [A+T+] | -0.04 | -0.07 –   -0.00 | **0.034** | -0.03 | -0.07 –  0.00 | 0.065 | -0.05 | -0.09 –   -0.01 | **0.008** | -0.04 | -0.08 –  -0.00 | **0.033** |
| **Random Effects** | | | | | | | | | | | | |
| σ^2^ | 0.49 | | | 0.42 | | | 0.31 | | | 0.29 | | |
| τ_00_ | 0.36 _WRAPNo_ | | | 0.42 _WRAPNo_ | | | 0.45 _WRAPNo_ | | | 0.49 _WRAPNo_ | | |
| τ_11_ |  | | |  | | | 0.00 _WRAPNo.c_curage_ | | |  | | |
| ρ_01_ |  | | |  | | | 0.22 _WRAPNo_ | | |  | | |
| ICC | 0.42 | | | 0.50 | | | 0.63 | | | 0.63 | | |
| N | 397 _WRAPNo_ | | | 397 _WRAPNo_ | | | 397 _WRAPNo_ | | | 397 _WRAPNo_ | | |
| Observations | 1970 | | | 1971 | | | 2001 | | | 2001 | | |
| Marginal R^2^ / Conditional R^2^ | 0.162 / 0.514 | | | 0.163 / 0.583 | | | 0.193 / 0.699 | | | 0.213 / 0.708 | | |

Linear-mixed effects models of logical memory and proper names subscores. PN=Proper Names, LM=Logical Memory (Total Score), PE=Practice Effects (nvis-1 operationalization utilized), A/T=Amyloid/Tau status (+=elevated, -=non-elevated). A-T- was the reference group for A/T status. Age was centered at 63.13 (sd=7.70). Bolded p-values meeting significance threshold of p<0.05.

**Supplemental Table 5. Aim 3: Sensitivity analysis AgexA/T status – Fluency Tasks**

|  | **Animal Naming** | | | **Letter Fluency (CFL)** | | |
| --- | --- | --- | --- | --- | --- | --- |
| *Predictors* | *β* | *CI* | *p* | *β* | *CI* | *p* |
| (Intercept) | -2.47 | -3.36 –  -1.57 | **<0.001** | -3.68 | -4.63 –   -2.72 | **<0.001** |
| Age (centered) | -0.03 | -0.04 –  -0.02 | **<0.001** | -0.02 | -0.03 –  -0.01 | **0.007** |
| Gender [Female] | -0.25 | -0.41 –  -0.09 | **0.003** | -0.00 | -0.17 –  0.17 | 0.988 |
| WRAT-III Reading Score | 0.02 | 0.02 – 0.03 | **<0.001** | 0.03 | 0.02 – 0.04 | **<0.001** |
| PE (nvis-1) | 0.04 | -0.00 –  0.09 | 0.056 | 0.10 | 0.06 – 0.14 | **<0.001** |
| A/T status [A+T-] | 0.18 | -0.07 –  0.43 | 0.157 | -0.17 | -0.47 –   0.13 | 0.259 |
| A/T status [A-T+] | -0.14 | -0.54 –  0.26 | 0.497 | -0.10 | -0.51 –  0.32 | 0.645 |
| A/T status [A+T+] | 0.01 | -0.24 –   0.27 | 0.922 | 0.27 | -0.02 –   0.57 | 0.069 |
| Age (centered)^2 |  |  |  | -0.00 | -0.00 –   -0.00 | **0.005** |
| PE × A/T status [A+T-] | 0.12 | 0.00 – 0.23 | **0.047** | 0.17 | 0.07 – 0.27 | **0.001** |
| PE × A/T status [A-T+] | -0.08 | -0.28 –  0.11 | 0.398 | -0.12 | -0.28 –  0.05 | 0.169 |
| PE × A/T status [A+T+] | 0.01 | -0.11 –   0.13 | 0.819 | -0.07 | -0.18 –  0.05 | 0.247 |
| Age (centered) × A/T status [A+T-] | -0.02 | -0.06 –   0.01 | 0.201 | -0.06 | -0.10 –   -0.02 | **0.002** |
| Age (centered) × A/T status [A-T+] | 0.02 | -0.04 – 0.08 | 0.581 | 0.03 | -0.03 –  0.08 | 0.388 |
| Age (centered) × A/T status [A+T+] | -0.05 | -0.08 –   -0.01 | **0.010** | -0.00 | -0.04 –  0.04 | 0.923 |
| **Random Effects** | | | | | | |
| σ^2^ | 0.35 | | | 0.24 | | |
| τ_00_ | 0.48 _WRAPNo_ | | | 0.61 _WRAPNo_ | | |
| ICC | 0.58 | | | 0.71 | | |
| N | 396 _WRAPNo_ | | | 397 _WRAPNo_ | | |
| Observations | 1567 | | | 1998 | | |
| Marginal R^2^ / Conditional R^2^ | 0.141 / 0.636 | | | 0.130 / 0.750 | | |

Linear-mixed effects models of fluency scores. PE=Practice Effects ( nvis-1 operationalization utilized), A/T=Amyloid/Tau status (+=elevated, -=non-elevated). A-T- was the reference group for A/T status. Age was centered at 63.13 (sd=7.70). Bolded p-values meeting significance threshold of p<0.05.

**Supplemental Figure 2. Aim 3 Sensitivity Models**


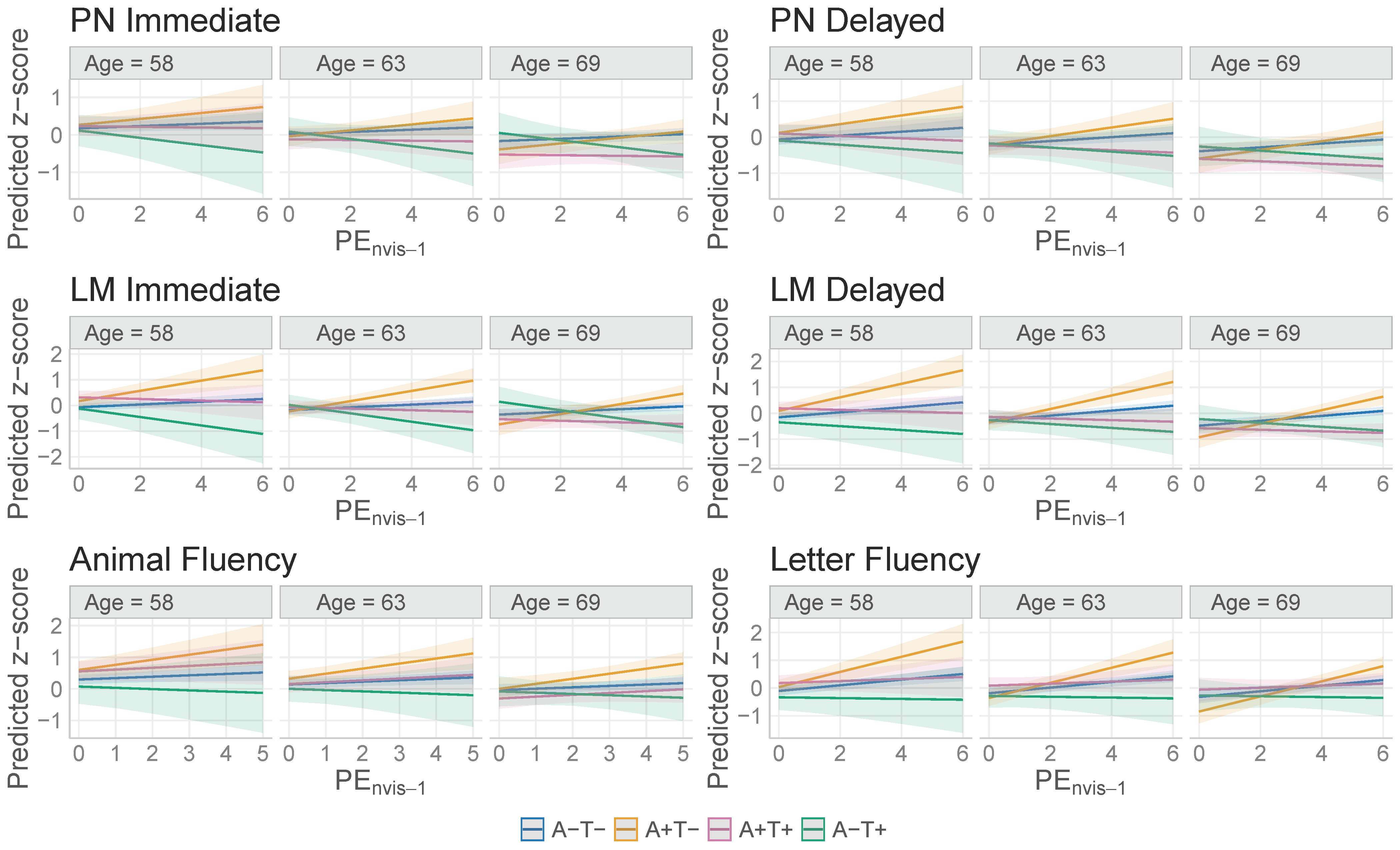


Interaction plots from linear mixed effects models presented in Supplemental Tables 3 (PN+LM, n=397) and 4 (fluency tasks, n=396), illustrating 3-way interaction: PE x A/T status x age. Ages displayed:1^st^ quartile=58, Mean age=63, 2^nd^ quartile=69. PN=Proper Name recall, LM=Logical Memory total score, PE_nvis-1_=Practice Effects (nvis-1 operationalization). A+=elevated amyloid PET, A-=non-elevated amyloid PET, T+=elevated tau PET, T-=non-elevated tau PET. A-T- (n=266) served as the reference group. Sample sizes for A/T groups: A+T- (n=57), A-T+ (n=20), and A+T+ (n=54).

**Supplementary Code:**

##Packages

library(dplyr)

library(ggplot2)

library(sjPlot)

library(lme4)

library(table1)

library(car)

##Factor the factors:

names(p)

p$gender <- as.factor(p$gender)

p$race <- as.factor(p$race)

p$apoe_bin <- as.factor(p$apoe_bin)

p$apoe_sum <- as.factor(p$apoe_sum)

p$pib_bin <- as.factor(p$pib_bin)

p$tau_bin <- as.factor(p$tau_bin)

###########################

##Table 1

table1(~ curage + factor(gender) + factor(race1) + factor(apoe_bin) + factor(FamilyHistory)+ factor(apoe_bin) + readstn

+ pib_age + mk_age | AT_bin, data=baseline)

table1(~ curage + factor(gender) + factor(race1) + factor(apoe_bin) + factor(FamilyHistory)+ factor(apoe_bin) + readstn

+ pib_age + mk_age | AT_bin, data=baseline.1)

baseline.1 <- filter(baseline, AT_bin != "")

t.test(curage~pib_bin, data=baseline)

chisq.test(table(baseline$gender, baseline$pib_bin))

chisq.test(table(baseline$race1, baseline$pib_bin))

chisq.test(table(baseline$FamilyHistory, baseline$pib_bin))

chisq.test(table(baseline$apoe_bin, baseline$pib_bin))

t.test(readstn~pib_bin, data=baseline)

t.test(pib_age~pib_bin, data=baseline)

summary(aov(curage~AT_bin, data=baseline.1))

chisq.test(table(baseline.1$gender, baseline.1$AT_bin))

chisq.test(table(baseline.1$race1, baseline.1$AT_bin))

chisq.test(table(baseline.1$FamilyHistory, baseline.1$AT_bin))

chisq.test(table(baseline.1$apoe_bin, baseline.1$AT_bin))

summary(aov(readstn~AT_bin, data=baseline.1))

summary(aov(mk_age~AT_bin, data=baseline.1))

################################

##Model building:

#############################################

##Animal Naming:

#############################################

p$an_pe_dif_base_bin <- as.factor(p$an_pe_dif_base_bin)

p$an_pe_dif_btwn_vis <- as.factor(p$an_pe_dif_btwn_vis)

p$an_pe_prior_vis <- as.factor(p$an_pe_prior_vis)

##lm models, just prac effects:

ani_mod.01 <- lm(Zanim ~ an_pe.1, data=p)

ani_mod.02 <- lm(Zanim ~ an_pe_dif_base_bin, data=p)

ani_mod.03 <- lm(Zanim ~ an_pe_dif_btwn_vis, data=p)

ani_mod.04 <- lm(Zanim ~ an_pe_prior_vis, data=p)

ani_mod.05 <- lm(Zanim ~ an_pe_dim_ret, data=p)

tab_model(ani_mod.01, ani_mod.02, ani_mod.03)

tab_model(ani_mod.04, ani_mod.05)

tab_model(ani_mod.01,ani_mod.02, ani_mod.03, ani_mod.04, ani_mod.05)

AIC(ani_mod.01, ani_mod.02, ani_mod.03, ani_mod.04, ani_mod.05)

##lm models, just covariates

ani_mod.1 <- lm(Zanim ~ c_curage + gender + readstn + an_pe.1, data=p)

ani_mod.2 <- lm(Zanim ~ c_curage + gender + readstn + an_pe_dif_base_bin, data=p)

ani_mod.3 <- lm(Zanim ~ c_curage + gender + readstn + an_pe_dif_btwn_vis, data=p)

ani_mod.4 <- lm(Zanim ~ c_curage + gender + readstn + an_pe_prior_vis, data=p)

ani_mod.5 <- lm(Zanim ~ c_curage + gender + readstn + an_pe_dim_ret, data=p)

tab_model(ani_mod.1, ani_mod.2, ani_mod.3)

tab_model(ani_mod.4, ani_mod.5)

AIC(ani_mod.1, ani_mod.2, ani_mod.3, ani_mod.4, ani_mod.5)

##lme models

ani_lme.01 <- lmer(Zanim ~ (1|WRAPNo), data=p, REML=FALSE)

ani_lme.02 <- lmer(Zanim ~ (c_curage|WRAPNo), data=p, REML=FALSE)

tab_model(ani_lme.01, ani_lme.02)

AIC(ani_lme.01)

AIC(ani_lme.02)

##Lower AIC for random slope & int, better model to build upon

p <- p %>%

rowwise() %>%

mutate(quad_age = (c_curage*c_curage))

summary(p$quad_age)

vif(ani_lme.3)

ani_lme.0 <- lmer(Zanim ~ c_curage + gender + readstn + (1|WRAPNo), data=p, REML=FALSE)

ani_lme.00 <- lmer(Zanim ~ c_curage + gender + readstn + (1|WRAPNo) + quad_age, data=p, REML=FALSE)

ani_lme.1 <- lmer(Zanim ~ c_curage + gender + readstn + (1|WRAPNo) + an_pe.1, data=p, REML=FALSE)

ani_lme.2<- lmer(Zanim ~ c_curage + gender + readstn + (1|WRAPNo) + an_pe_dif_base_bin, data=p, REML=FALSE)

ani_lme.3 <- lmer(Zanim ~ c_curage + gender + readstn + (1|WRAPNo) + an_pe_dif_btwn_vis, data=p, REML=FALSE)

ani_lme.4<- lmer(Zanim ~ c_curage + gender + readstn + (1|WRAPNo) + an_pe_prior_vis, data=p, REML=FALSE)

ani_lme.5 <- lmer(Zanim ~ c_curage + gender + readstn + (1|WRAPNo) + an_pe_dim_ret, data=p, REML=FALSE)

tab_model(ani_lme.1, ani_lme.2, ani_lme.3)

tab_model(ani_lme.4, ani_lme.5)

ab_model(ani_lme.4, ani_lme.5)

tab_model(ani_mod.01, ani_mod.05, ani_mod.1, ani_mod.5)

summary(ani_lme.00)

tab_model(ani_lme.00)

tab_model(ani_lme.0, ani_lme.00)

AIC(ani_lme.0)

AIC(ani_lme.00)

#############################################

##cfl:

#############################################

p$flu_pe_dif_base_bin <- as.factor(p$flu_pe_dif_base_bin)

p$flu_pe_dif_btwn_vis <- as.factor(p$flu_pe_dif_btwn_vis)

p$flu_pe_prior_vis <- as.factor(p$flu_pe_prior_vis)

##lm models, just prac effects:

cfl_mod.01 <- lm(Zflucfl ~ flu_pe.1, data=p)

cfl_mod.02 <- lm(Zflucfl ~ flu_pe_dif_base_bin, data=p)

cfl_mod.03 <- lm(Zflucfl ~ flu_pe_dif_btwn_vis, data=p)

cfl_mod.04 <- lm(Zflucfl ~ flu_pe_prior_vis, data=p)

cfl_mod.05 <- lm(Zflucfl ~ flu_pe_dim_ret, data=p)

tab_model(cfl_mod.01, cfl_mod.02, cfl_mod.03, cfl_mod.04, cfl_mod.05)

AIC(cfl_mod.01)

AIC(cfl_mod.02)

AIC(cfl_mod.03)

##lm models, just covariates

cfl_mod.1 <- lm(Zflucfl ~ c_curage + gender + readstn + flu_pe.1, data=p)

cfl_mod.2 <- lm(Zflucfl ~ c_curage + gender + readstn + flu_pe_dif_base_bin, data=p)

cfl_mod.3 <- lm(Zflucfl ~ c_curage + gender + readstn + flu_pe_dif_btwn_vis, data=p)

cfl_mod.4 <- lm(Zflucfl ~ c_curage + gender + readstn + flu_pe_prior_vis, data=p)

cfl_mod.5 <- lm(Zflucfl ~ c_curage + gender + readstn + flu_pe_dim_ret, data=p)

tab_model(cfl_mod.1, cfl_mod.2, cfl_mod.3, cfl_mod.4, cfl_mod.5)

AIC(cfl_mod.1)

AIC(cfl_mod.2)

AIC(cfl_mod.3)

##lme models

cfl_lme.01 <- lmer(Zflucfl ~ (1|WRAPNo), data=p, REML=FALSE)

cfl_lme.02 <- lmer(Zflucfl ~ (c_curage|WRAPNo), data=p, REML=FALSE)

tab_model(cfl_lme.01, cfl_lme.02)

AIC(cfl_lme.01)

AIC(cfl_lme.02)

##Convergence issues for random slope & int, just int likely better model to build upon

vif(ani_lme.1)

cfl_lme.0 <- lmer(Zflucfl ~ c_curage + gender + readstn + (1|WRAPNo), data=p, REML=FALSE)

cfl_lme.00 <- lmer(Zflucfl ~ c_curage + gender + readstn + (1|WRAPNo) + quad_age, data=p, REML=FALSE)

cfl_lme.1 <- lmer(Zflucfl ~ c_curage + gender + readstn + (1|WRAPNo) + flu_pe.1, data=p, REML=FALSE)

cfl_lme.2<- lmer(Zflucfl ~ c_curage + gender + readstn + (1|WRAPNo) + flu_pe_dif_base_bin, data=p, REML=FALSE)

cfl_lme.3 <- lmer(Zflucfl ~ c_curage + gender + readstn + (1|WRAPNo) + flu_pe_dif_btwn_vis, data=p, REML=FALSE)

cfl_lme.4 <- lmer(Zflucfl ~ c_curage + gender + readstn + (1|WRAPNo) + flu_pe_prior_vis, data=p, REML=FALSE)

cfl_lme.5 <- lmer(Zflucfl ~ c_curage + gender + readstn + (1|WRAPNo) + flu_pe_dim_ret, data=p, REML=FALSE)

tab_model(ani_lme.1, ani_lme.3)

##Singularity issues with mod 2

summary(ani_lme.00)

tab_model(ani_lme.00)

tab_model(ani_lme.0, ani_lme.00)

AIC(ani_lme.0)

AIC(ani_lme.00)

############################################

##ImmAB:

#############################################

p$immab_pe_dif_base_bin <- as.factor(p$immab_pe_dif_base_bin)

p$immab_pe_dif_btwn_vis <- as.factor(p$immab_pe_dif_btwn_vis)

p$lm_pe_prior_vis <- as.factor(p$lm_pe_prior_vis)

##lm models, just prac effects:

iab_mod.01 <- lm(ZPN_ImmAB ~ lm_pe.1, data=p)

iab_mod.02 <- lm(ZPN_ImmAB ~ immab_pe_dif_base_bin, data=p)

iab_mod.03 <- lm(ZPN_ImmAB ~ immab_pe_dif_btwn_vis, data=p)

iab_mod.04 <- lm(ZPN_ImmAB ~ lm_pe_prior_vis, data=p)

iab_mod.05 <- lm(ZPN_ImmAB ~ lm_pe_dim_ret, data=p)

tab_model(iab_mod.01, iab_mod.02, iab_mod.03)

##lm models, just covariates

iab_mod.1 <- lm(ZPN_ImmAB ~ c_curage + gender + readstn + lm_pe.1, data=p)

iab_mod.2 <- lm(ZPN_ImmAB ~ c_curage + gender + readstn + immab_pe_dif_base_bin, data=p)

iab_mod.3 <- lm(ZPN_ImmAB ~ c_curage + gender + readstn + immab_pe_dif_btwn_vis, data=p)

iab_mod.4 <- lm(ZPN_ImmAB ~ c_curage + gender + readstn + lm_pe_prior_vis, data=p)

iab_mod.5 <- lm(ZPN_ImmAB ~ c_curage + gender + readstn + lm_pe_dim_ret, data=p)

tab_model(iab_mod.1, iab_mod.2, iab_mod.3)

##lme models

iab_lme.01 <- lmer(ZPN_ImmAB ~ (1|WRAPNo), data=p, REML=FALSE)

iab_lme.02 <- lmer(ZPN_ImmAB ~ (c_curage|WRAPNo), data=p, REML=FALSE)

tab_model(iab_lme.01, iab_lme.02)

AIC(iab_lme.01)

AIC(iab_lme.02)

##Lower AIC for random int, better model to build upon

vif(ani_lme.3)

iab_lme.0 <- lmer(ZPN_ImmAB ~ c_curage + gender + readstn + (1|WRAPNo), data=p, REML=FALSE)

iab_lme.00 <- lmer(ZPN_ImmAB ~ c_curage + gender + readstn + (1|WRAPNo) + quad_age, data=p, REML=FALSE)

iab_lme.1 <- lmer(ZPN_ImmAB ~ c_curage + gender + readstn + (1|WRAPNo) + lm_pe.1, data=p, REML=FALSE)

iab_lme.2<- lmer(ZPN_ImmAB ~ c_curage + gender + readstn + (1|WRAPNo) + immab_pe_dif_base_bin, data=p, REML=FALSE)

iab_lme.3 <- lmer(ZPN_ImmAB ~ c_curage + gender + readstn + (1|WRAPNo) + immab_pe_dif_btwn_vis, data=p, REML=FALSE)

iab_lme.4 <- lmer(ZPN_ImmAB ~ c_curage + gender + readstn + (1|WRAPNo) + lm_pe_prior_vis, data=p, REML=FALSE)

iab_lme.5 <- lmer(ZPN_ImmAB ~ c_curage + gender + readstn + (1|WRAPNo) + lm_pe_dim_ret, data=p, REML=FALSE)

tab_model(iab_lme.1, iab_lme.3)

##Singularity issues with mod 2

summary(iab_lme.00)

tab_model(iab_lme.00)

tab_model(iab_lme.0, iab_lme.00)

AIC(iab_lme.0)

AIC(iab_lme.00)

#############################################

##DelAB:

#############################################

p$delab_pe_dif_base_bin <- as.factor(p$delab_pe_dif_base_bin)

p$delab_pe_dif_btwn_vis <- as.factor(p$delab_pe_dif_btwn_vis)

##lm models, just prac effects:

dab_mod.01 <- lm(ZPN_DelAB ~ lm_pe.1, data=p)

dab_mod.02 <- lm(ZPN_DelAB ~ delab_pe_dif_base_bin, data=p)

dab_mod.03 <- lm(ZPN_DelAB ~ delab_pe_dif_btwn_vis, data=p)

dab_mod.04 <- lm(ZPN_DelAB ~ lm_pe_prior_vis, data=p)

dab_mod.05 <- lm(ZPN_DelAB ~ lm_pe_dim_ret, data=p)

tab_model(dab_mod.01, dab_mod.02, dab_mod.03)

##lm models, just covariates

dab_mod.1 <- lm(ZPN_DelAB ~ c_curage + gender + readstn + lm_pe.1, data=p)

dab_mod.2 <- lm(ZPN_DelAB ~ c_curage + gender + readstn + delab_pe_dif_base_bin, data=p)

dab_mod.3 <- lm(ZPN_DelAB ~ c_curage + gender + readstn + delab_pe_dif_btwn_vis, data=p)

dab_mod.4 <- lm(ZPN_DelAB ~ c_curage + gender + readstn + lm_pe_prior_vis, data=p)

dab_mod.5 <- lm(ZPN_DelAB ~ c_curage + gender + readstn + lm_pe_dim_ret, data=p)

tab_model(dab_mod.1, dab_mod.2, dab_mod.3)

##lme models

dab_lme.01 <- lmer(ZPN_DelAB ~ (1|WRAPNo), data=p, REML=FALSE)

dab_lme.02 <- lmer(ZPN_DelAB ~ (c_curage|WRAPNo), data=p, REML=FALSE)

tab_model(dab_lme.01, dab_lme.02)

AIC(dab_lme.01)

AIC(dab_lme.02)

##Lower AIC for random int, better model to build upon

vif(ani_lme.3)

dab_lme.0 <- lmer(ZPN_DelAB ~ c_curage + gender + readstn + (1|WRAPNo), data=p, REML=FALSE)

dab_lme.00 <- lmer(ZPN_DelAB ~ c_curage + gender + readstn + (1|WRAPNo) + quad_age, data=p, REML=FALSE)

dab_lme.1 <- lmer(ZPN_DelAB ~ c_curage + gender + readstn + (1|WRAPNo) + lm_pe.1, data=p, REML=FALSE)

dab_lme.2<- lmer(ZPN_DelAB ~ c_curage + gender + readstn + (1|WRAPNo) + delab_pe_dif_base_bin, data=p, REML=FALSE)

dab_lme.3 <- lmer(ZPN_DelAB ~ c_curage + gender + readstn + (1|WRAPNo) + delab_pe_dif_btwn_vis, data=p, REML=FALSE)

dab_lme.4 <- lmer(ZPN_DelAB ~ c_curage + gender + readstn + (1|WRAPNo) + lm_pe_prior_vis, data=p, REML=FALSE)

dab_lme.5 <- lmer(ZPN_DelAB ~ c_curage + gender + readstn + (1|WRAPNo) + lm_pe_dim_ret, data=p, REML=FALSE)

tab_model(dab_lme.1, dab_lme.3)

##Singularity issues with mod 2

summary(dab_lme.00)

tab_model(dab_lme.00)

tab_model(dab_lme.0, dab_lme.00)

AIC(dab_lme.0)

AIC(dab_lme.00)

#################################

##Main Aim models:

##Center age:

p$c_curage <- scale(p$curage, center = TRUE, scale = FALSE)

p <- mutate(p, AT_bin = ifelse(pib_bin == 0 & tau_bin == 0, 0,

ifelse(pib_bin == 1 & tau_bin == 0, 1,

ifelse(pib_bin == 1 & tau_bin == 1, 2, 4))))

p$AT_bin <- as.factor(p$AT_bin)

#############################################

##Animal Naming:

#############################################

##amyloid only###########################

ani_lme.1 <- lmer(Zanim ~ c_curage + gender + readstn + (1|WRAPNo) + trt +

an_pe.1*pib_bin, data=p, REML=FALSE)

tab_model(ani_lme.1)

vif(ani_lme.1)

##ADD TAU:################################

ani_lme.1.1 <- lmer(Zanim ~ c_curage + gender + readstn + (1|WRAPNo) + trt +

an_pe.1*AT_bin, data=p, REML=FALSE)

tab_model(ani_lme.1.1)

vif(ani_lme.1.1)

##Add age:

ani_lme.1.2 <- lmer(Zanim ~ c_curage + gender + readstn + (1|WRAPNo) + trt +

an_pe.1*AT_bin + c_curage*AT_bin, data=p, REML=FALSE)

tab_model(ani_lme.1.2)

vif(ani_lme.1.2)

##Simple slopes:

an.1 <- emtrends(ani_lme.1.1, var = "an_pe.1",

at = list(AT_bin = c("0", "1", "2", "3")),

lmer.df = "satterthwaite")

summary(an.1, infer=TRUE)

an.2 <- emtrends(ani_lme.1.2, var = "an_pe.1",

at = list(AT_bin = c("0", "1", "2", "3")),

lmer.df = "satterthwaite")

summary(an.2, infer=TRUE)

an.3 <- emtrends(ani_lme.1.2, var = "c_curage",

at = list(AT_bin = c("0", "1", "2", "3")),

lmer.df = "satterthwaite")

summary(an.3, infer=TRUE)

#############################################

##CFL:

#############################################

##Amyloid:

cfl_lme.1 <- lmer(Zflucfl ~ c_curage + gender + readstn + (1|WRAPNo) + I(c_curage^2)+ trt +

cfl_pe.1*pib_bin, data=p, REML=FALSE)

tab_model(cfl_lme.1)

##Aim 2: fluency models

tab_model(ani_lme.1, cfl_lme.1)

##ADD TAU:

cfl_lme.1.1 <- lmer(Zflucfl ~ c_curage + gender + readstn + (1|WRAPNo) + I(c_curage^2) + trt +

cfl_pe.1*AT_bin, data=p, REML=FALSE)

tab_model(cfl_lme.1.1)

##Aim 3: fluency models

tab_model(ani_lme.1.1, cfl_lme.1.1)

vif(cfl_lme.1.1)

##Simple slopes:

lf.1 <- emtrends(cfl_lme.1.1, var = "cfl_pe.1",

at = list(AT_bin = c("0", "1", "2", "3")),

lmer.df = "satterthwaite")

summary(lf.1, infer=TRUE)

emm2 <- emmeans(q2, ~ pib_gender * c_age) #Logical Memory

contrasts <- list(

`cont` = c(Female_Pos = 1, Female_Neg = 0, Male_Pos = -1, Male_Neg = 0)

)

comp2 <- contrast(emm2, contrasts)

summary(comp2)

##Add age*AT bin:

cfl_lme.1.2 <- lmer(Zflucfl ~ c_curage + gender + readstn + (1|WRAPNo) + I(c_curage^2) + trt +

cfl_pe.1*AT_bin +

c_curage*AT_bin, data=p, REML=FALSE)

vif(cfl_lme.1.2)

tab_model(ani_lme.1.2, cfl_lme.1.2)

lf.2 <- emtrends(cfl_lme.1.2, var = "cfl_pe.1",

at = list(AT_bin = c("0", "1", "2", "3")),

lmer.df = "satterthwaite")

summary(lf.2, infer=TRUE)

lf.3 <- emtrends(cfl_lme.1.2, var = "c_curage",

at = list(AT_bin = c("0", "1", "2", "3")),

lmer.df = "satterthwaite")

summary(lf.3, infer=TRUE)

############################################

##Logical Memory (1)

############################################

##Aim 2:

wtot1_lme.1 <- lmer(ZwmsrTot ~ c_curage + gender + readstn + (c_curage|WRAPNo) + I(c_curage^2) +

lm_pe.1*pib_bin, data=p, REML=FALSE)

tab_model(wtot1_lme.1)

##Simple slopes:

lmi.1 <- emtrends(wtot1_lme.1, var = "lm_pe.1",

at = list(pib_bin = c("0", "1")),

lmer.df = "satterthwaite")

summary(lmi.1, infer=TRUE)

#Add age:

wtot1_lme.2 <- lmer(ZwmsrTot ~ c_curage + gender + readstn + (c_curage|WRAPNo) + I(c_curage^2) +

lm_pe.1*pib_bin +

c_curage*pib_bin, data=p, REML=FALSE)

tab_model(wtot1_lme.2)

##Simple slopes:

lmi.2 <- emtrends(wtot1_lme.2, var = "lm_pe.1",

at = list(pib_bin = c("0", "1")),

lmer.df = "satterthwaite")

summary(lmi.2, infer=TRUE)

lmi.2 <- emtrends(wtot1_lme.2, var = "c_curage",

at = list(pib_bin = c("0", "1")),

lmer.df = "satterthwaite")

summary(lmi.2, infer=TRUE)

##Aim 3 - Add Tau:

wtot1_lme.1.1 <- lmer(ZwmsrTot ~ c_curage + gender + readstn + (c_curage|WRAPNo) + I(c_curage^2)

+ lm_pe.1*AT_bin, data=p, REML=FALSE)

tab_model(wtot1_lme.1.1)

##Simple slopes:

lm.2 <- emtrends(wtot1_lme.1.1, var = "lm_pe.1",

at = list(AT_bin = c("0", "1", "2", "3")),

lmer.df = "satterthwaite")

summary(lm.2, infer=TRUE)

##Add age:

wtot1_lme.1.2 <- lmer(ZwmsrTot ~ c_curage + gender + readstn + (c_curage|WRAPNo) + I(c_curage^2)

+ lm_pe.1*AT_bin +c_curage*AT_bin

, data=p, REML=FALSE)

tab_model(wtot1_lme.1.2)

##Simple slopes:

lm.3 <- emtrends(wtot1_lme.1.2, var = "lm_pe.1",

at = list(AT_bin = c("0", "1", "2", "3")),

lmer.df = "satterthwaite")

summary(lm.3, infer=TRUE)

lm.4 <- emtrends(wtot1_lme.1.2, var = "c_curage",

at = list(AT_bin = c("0", "1", "2", "3")),

lmer.df = "satterthwaite")

summary(lm.4, infer=TRUE)

############################################

##Logical Memory (2)

############################################

##Aim 2:

wtot2_lme.1 <- lmer(ZwmsrTot2 ~ c_curage + gender + readstn + (1|WRAPNo) + I(c_curage^2) +

lm_pe.1*pib_bin, data=p, REML=FALSE)

tab_model(wtot2_lme.1)

vif(wtot2_lme.1)

tab_model(wtot1_lme.1, wtot2_lme.1)

##Simple slopes:

lmd.1 <- emtrends(wtot2_lme.1, var = "lm_pe.1",

at = list(pib_bin = c("0", "1")),

lmer.df = "satterthwaite")

summary(lmd.1, infer=TRUE)

##Add age:

wtot2_lme.2 <- lmer(ZwmsrTot2 ~ c_curage + gender + readstn + (1|WRAPNo) + I(c_curage^2) +

lm_pe.1*pib_bin +

c_curage*pib_bin, data=p, REML=FALSE)

tab_model(wtot2_lme.2)

##Simple slopes:

lmd.2 <- emtrends(wtot2_lme.2, var = "lm_pe.1",

at = list(pib_bin = c("0", "1")),

lmer.df = "satterthwaite")

summary(lmd.2, infer=TRUE)

lmd.3 <- emtrends(wtot2_lme.2, var = "c_curage",

at = list(pib_bin = c("0", "1")),

lmer.df = "satterthwaite")

summary(lmd.3, infer=TRUE)

##Aim 3: ADD TAU:

wtot_lme.1.1 <- lmer(ZwmsrTot2 ~ c_curage + gender + readstn + (1|WRAPNo) + I(c_curage^2)

+ lm_pe.1*AT_bin, data=p, REML=FALSE)

tab_model(wtot_lme.1.1)

tab_model(wtot1_lme.1.1, wtot_lme.1.1)

##Simple slopes:

lm.2 <- emtrends(wtot_lme.1.1, var = "lm_pe.1",

at = list(AT_bin = c("0", "1", "2", "3")),

lmer.df = "satterthwaite")

summary(lm.2, infer=TRUE)

##Add age:

wtot_lme.1.2 <- lmer(ZwmsrTot2 ~ c_curage + gender + readstn + (1|WRAPNo) + I(c_curage^2)

+ lm_pe.1*AT_bin + c_curage*AT_bin, data=p, REML=FALSE)

tab_model(wtot_lme.1.2)

##Simple slopes:

lm.3 <- emtrends(wtot_lme.1.2, var = "lm_pe.1",

at = list(AT_bin = c("0", "1", "2", "3")),

lmer.df = "satterthwaite")

summary(lm.3, infer=TRUE)

lm.4 <- emtrends(wtot_lme.1.2, var = "c_curage",

at = list(AT_bin = c("0", "1", "2", "3")),

lmer.df = "satterthwaite")

summary(lm.4, infer=TRUE)

########################################

##Proper Names Immediate AB

#########################################

##convergence issues - removed c_curage|WRAPNo for rand int only

immab_lme.1 <- lmer(ZPN_ImmAB ~ c_curage + gender + readstn + (1|WRAPNo) +

pn_pe.1*pib_bin, data=p, REML=FALSE)

tab_model(immab_lme.1)

##Simple slopes:

pn.1 <- emtrends(immab_lme.1, var = "pn_pe.1",

at = list(pib_bin = c("0", "1")),

lmer.df = "satterthwaite")

summary(pn.1, infer=TRUE)

##ADD AGE*pe

immab_lme.2 <- lmer(ZPN_ImmAB ~ c_curage + gender + readstn + (1|WRAPNo) + pn_pe.1*pib_bin +

pib_bin*c_curage, data=p, REML=FALSE)

tab_model(immab_lme.2)

##Simple slopes:

pn.2 <- emtrends(immab_lme.2, var = "pn_pe.1",

at = list(pib_bin = c("0", "1")),

lmer.df = "satterthwaite")

summary(pn.2, infer=TRUE)

pn.3 <- emtrends(immab_lme.2, var = "c_curage",

at = list(pib_bin = c("0", "1")),

lmer.df = "satterthwaite")

summary(pn.3, infer=TRUE)

##Aim 3: ADD TAU:

immab_lme.1.1 <- lmer(ZPN_ImmAB ~ c_curage + gender + readstn + (1|WRAPNo) + pn_pe.1*AT_bin, data=p, REML=FALSE)

tab_model(immab_lme.1.1)

##Simple slopes:

pn.4 <- emtrends(immab_lme.1.1, var = "pn_pe.1",

at = list(AT_bin = c("0", "1", "2", "3")),

lmer.df = "satterthwaite")

summary(pn.4, infer=TRUE)

##Add age:

immab_lme.1.2 <- lmer(ZPN_ImmAB ~ c_curage + gender + readstn + (1|WRAPNo) + pn_pe.1*AT_bin +

c_curage*AT_bin, data=p, REML=FALSE)

tab_model(immab_lme.1.2)

##Simple slopes:

pn.5 <- emtrends(immab_lme.1.2, var = "pn_pe.1",

at = list(AT_bin = c("0", "1", "2", "3")),

lmer.df = "satterthwaite")

summary(pn.5, infer=TRUE)

pn.6 <- emtrends(immab_lme.1.2, var = "c_curage",

at = list(AT_bin = c("0", "1", "2", "3")),

lmer.df = "satterthwaite")

summary(pn.6, infer=TRUE)

##############################################

##Proper Names Delayed AB

#############################################

##AMyloid:

delab_lme.1 <- lmer(ZPN_DelAB ~ c_curage + gender + readstn + (1|WRAPNo) +

pn_pe.1*pib_bin, data=p, REML=FALSE)

tab_model(delab_lme.1)

###Simple slopes:

pn.1 <- emtrends(delab_lme.1, var = "pn_pe.1",

at = list(pib_bin = c("0", "1")),

lmer.df = "satterthwaite")

summary(pn.1, infer=TRUE)

##Add age*pib

delab_lme.2 <- lmer(ZPN_DelAB ~ c_curage + gender + readstn + (1|WRAPNo) + pn_pe.1*pib_bin +

c_curage*pib_bin, data=p, REML=FALSE)

tab_model(delab_lme.2)

##Simple slopes:

pn.2 <- emtrends(delab_lme.2, var = "pn_pe.1",

at = list(pib_bin = c("0", "1")),

lmer.df = "satterthwaite")

summary(pn.2, infer=TRUE)

pn.3 <- emtrends(delab_lme.2, var = "c_curage",

at = list(pib_bin = c("0", "1")),

lmer.df = "satterthwaite")

summary(pn.3, infer=TRUE)

##ADD TAU:

delab_lme.1.1 <- lmer(ZPN_DelAB ~ c_curage + gender + readstn + (1|WRAPNo) +

pn_pe.1*AT_bin, data=p, REML=FALSE)

tab_model(delab_lme.1.1)

##Simple slopes:

pn.4 <- emtrends(delab_lme.1.1, var = "pn_pe.1",

at = list(AT_bin = c("0", "1", "2", "3")),

lmer.df = "satterthwaite")

summary(pn.4, infer=TRUE)

##Add age:

delab_lme.1.2 <- lmer(ZPN_DelAB ~ c_curage + gender + readstn + (1|WRAPNo) + pn_pe.1*AT_bin +

c_curage*AT_bin, data=p, REML=FALSE)

tab_model(delab_lme.1.2)

pn.5 <- emtrends(delab_lme.1.2, var = "pn_pe.1",

at = list(AT_bin = c("0", "1", "2", "3")),

lmer.df = "satterthwaite")

summary(pn.5, infer=TRUE)

pn.6 <- emtrends(delab_lme.1.2, var = "c_curage",

at = list(AT_bin = c("0", "1", "2", "3")),

lmer.df = "satterthwaite")

summary(pn.6, infer=TRUE)

###########################################

##LM/PN tab_models:

vif(delab_lme.1.2)

tab_model(immab_lme.1, delab_lme.1, wtot1_lme.1, wtot2_lme.1)

tab_model(wtot1_lme.2, wtot2_lme.2, immab_lme.2, delab_lme.2)

tab_model(wtot1_lme.1.1, wtot_lme.1.1, immab_lme.1.1, delab_lme.1.1)

tab_model(wtot1_lme.1.2, wtot_lme.1.2, immab_lme.1.2, delab_lme.1.2)

###########################################

##Sensitivity analyses

##re-run models with baseline age and time since baseline:

bl <- p %>%

group_by(WRAPNo) %>%

filter(curage == min(curage))

n_distinct(p$WRAPNo)

bl <- select(bl, WRAPNo, curage)

bl <- rename(bl, bl_age = curage)

p <- left_join(p, bl, by="WRAPNo")

p <- p %>%

rowwise() %>%

mutate(age_dif = (curage - bl_age))

############################################

##Logical Memory (1)

############################################

##Aim 2:

wtot1_lme.1 <- lmer(ZwmsrTot ~ bl_age + gender + readstn + (1|WRAPNo) + lm_pe.1*pib_bin, data=p, REML=FALSE)

tab_model(wtot1_lme.1)

vif(wtot1_lme.1)

##Simple slopes:

lmi.1 <- emtrends(wtot1_lme.1, var = "lm_pe.1",

at = list(pib_bin = c("0", "1")),

lmer.df = "satterthwaite")

summary(lmi.1, infer=TRUE)

#Add age:

wtot1_lme.2 <- lmer(ZwmsrTot ~ bl_age + gender + readstn + (1|WRAPNo) + lm_pe.1*pib_bin +

age_dif*pib_bin + bl_age*pib_bin, data=p, REML=FALSE)

tab_model(wtot1_lme.2)

vif(wtot1_lme.2)

##Simple slopes:

lmi.2 <- emtrends(wtot1_lme.2, var = "lm_pe.1",

at = list(pib_bin = c("0", "1")),

lmer.df = "satterthwaite")

summary(lmi.2, infer=TRUE)

lmi.2 <- emtrends(wtot1_lme.2, var = "c_curage",

at = list(pib_bin = c("0", "1")),

lmer.df = "satterthwaite")

summary(lmi.2, infer=TRUE)

##Aim 3 - Add Tau:

wtot1_lme.1.1 <- lmer(ZwmsrTot ~ c_curage + gender + readstn + (c_curage|WRAPNo) + I(c_curage^2) + lm_pe.1*AT_bin, data=p, REML=FALSE)

tab_model(wtot1_lme.1.1)

##Simple slopes:

lm.2 <- emtrends(wtot1_lme.1.1, var = "lm_pe.1",

at = list(AT_bin = c("0", "1", "2", "3")),

lmer.df = "satterthwaite")

summary(lm.2, infer=TRUE)

##Add age:

wtot1_lme.1.2 <- lmer(ZwmsrTot ~ c_curage + gender + readstn + (c_curage|WRAPNo) + I(c_curage^2) + lm_pe.1*AT_bin +c_curage*AT_bin

, data=p, REML=FALSE)

tab_model(wtot1_lme.1.2)

##Simple slopes:

lm.3 <- emtrends(wtot1_lme.1.2, var = "lm_pe.1",

at = list(AT_bin = c("0", "1", "2", "3")),

lmer.df = "satterthwaite")

summary(lm.3, infer=TRUE)

lm.4 <- emtrends(wtot1_lme.1.2, var = "c_curage",

at = list(AT_bin = c("0", "1", "2", "3")),

lmer.df = "satterthwaite")

summary(lm.4, infer=TRUE)

############################################

##Logical Memory (2)

############################################

##Aim 2:

wtot2_lme.1 <- lmer(ZwmsrTot2 ~ c_curage + gender + readstn + (1|WRAPNo) + I(c_curage^2) + lm_pe.1*pib_bin, data=p, REML=FALSE)

tab_model(wtot2_lme.1)

tab_model(wtot1_lme.1, wtot2_lme.1)

##Simple slopes:

lmd.1 <- emtrends(wtot2_lme.1, var = "lm_pe.1",

at = list(pib_bin = c("0", "1")),

lmer.df = "satterthwaite")

summary(lmd.1, infer=TRUE)

##Add age:

wtot2_lme.2 <- lmer(ZwmsrTot2 ~ c_curage + gender + readstn + (1|WRAPNo) + I(c_curage^2) + lm_pe.1*pib_bin +

c_curage*pib_bin, data=p, REML=FALSE)

tab_model(wtot2_lme.2)

##Simple slopes:

lmd.2 <- emtrends(wtot2_lme.2, var = "lm_pe.1",

at = list(pib_bin = c("0", "1")),

lmer.df = "satterthwaite")

summary(lmd.2, infer=TRUE)

lmd.3 <- emtrends(wtot2_lme.2, var = "c_curage",

at = list(pib_bin = c("0", "1")),

lmer.df = "satterthwaite")

summary(lmd.3, infer=TRUE)

##Aim 3: ADD TAU:

wtot_lme.1.1 <- lmer(ZwmsrTot2 ~ c_curage + gender + readstn + (1|WRAPNo) + I(c_curage^2)

+ lm_pe.1*AT_bin, data=p, REML=FALSE)

tab_model(wtot_lme.1.1)

tab_model(wtot1_lme.1.1, wtot_lme.1.1)

##Simple slopes:

lm.2 <- emtrends(wtot_lme.1.1, var = "lm_pe.1",

at = list(AT_bin = c("0", "1", "2", "3")),

lmer.df = "satterthwaite")

summary(lm.2, infer=TRUE)

##Add age:

wtot_lme.1.2 <- lmer(ZwmsrTot2 ~ c_curage + gender + readstn + (1|WRAPNo) + I(c_curage^2)

+ lm_pe.1*AT_bin + c_curage*AT_bin, data=p, REML=FALSE)

tab_model(wtot_lme.1.2)

##Simple slopes:

lm.3 <- emtrends(wtot_lme.1.2, var = "lm_pe.1",

at = list(AT_bin = c("0", "1", "2", "3")),

lmer.df = "satterthwaite")

summary(lm.3, infer=TRUE)

lm.4 <- emtrends(wtot_lme.1.2, var = "c_curage",

at = list(AT_bin = c("0", "1", "2", "3")),

lmer.df = "satterthwaite")

summary(lm.4, infer=TRUE)

########################################

##Proper Names Immediate AB

#########################################

##convergence issues - removed c_curage|WRAPNo for rand int only

immab_lme.1 <- lmer(ZPN_ImmAB ~ c_curage + gender + readstn + (1|WRAPNo) + pn_pe.1*pib_bin, data=p, REML=FALSE)

tab_model(immab_lme.1)

##Simple slopes:

pn.1 <- emtrends(immab_lme.1, var = "pn_pe.1",

at = list(pib_bin = c("0", "1")),

lmer.df = "satterthwaite")

summary(pn.1, infer=TRUE)

##ADD AGE*pe

immab_lme.2 <- lmer(ZPN_ImmAB ~ c_curage + gender + readstn + (1|WRAPNo) + pn_pe.1*pib_bin +

pib_bin*c_curage, data=p, REML=FALSE)

tab_model(immab_lme.2)

##Simple slopes:

pn.2 <- emtrends(immab_lme.2, var = "pn_pe.1",

at = list(pib_bin = c("0", "1")),

lmer.df = "satterthwaite")

summary(pn.2, infer=TRUE)

pn.3 <- emtrends(immab_lme.2, var = "c_curage",

at = list(pib_bin = c("0", "1")),

lmer.df = "satterthwaite")

summary(pn.3, infer=TRUE)

##Aim 3: ADD TAU:

immab_lme.1.1 <- lmer(ZPN_ImmAB ~ c_curage + gender + readstn + (1|WRAPNo) + pn_pe.1*AT_bin, data=p, REML=FALSE)

tab_model(immab_lme.1.1)

##Simple slopes:

pn.4 <- emtrends(immab_lme.1.1, var = "pn_pe.1",

at = list(AT_bin = c("0", "1", "2", "3")),

lmer.df = "satterthwaite")

summary(pn.4, infer=TRUE)

##Add age:

immab_lme.1.2 <- lmer(ZPN_ImmAB ~ c_curage + gender + readstn + (1|WRAPNo) + pn_pe.1*AT_bin +

c_curage*AT_bin, data=p, REML=FALSE)

tab_model(immab_lme.1.2)

##Simple slopes:

pn.5 <- emtrends(immab_lme.1.2, var = "pn_pe.1",

at = list(AT_bin = c("0", "1", "2", "3")),

lmer.df = "satterthwaite")

summary(pn.5, infer=TRUE)

pn.6 <- emtrends(immab_lme.1.2, var = "c_curage",

at = list(AT_bin = c("0", "1", "2", "3")),

lmer.df = "satterthwaite")

summary(pn.6, infer=TRUE)

##############################################

##Proper Names Delayed AB

#############################################

##AMyloid:

delab_lme.1 <- lmer(ZPN_DelAB ~ c_curage + gender + readstn + (1|WRAPNo) + pn_pe.1*pib_bin, data=p, REML=FALSE)

tab_model(delab_lme.1)

###Simple slopes:

pn.1 <- emtrends(delab_lme.1, var = "pn_pe.1",

at = list(pib_bin = c("0", "1")),

lmer.df = "satterthwaite")

summary(pn.1, infer=TRUE)

##Add age*pib

delab_lme.2 <- lmer(ZPN_DelAB ~ c_curage + gender + readstn + (1|WRAPNo) + pn_pe.1*pib_bin +

c_curage*pib_bin, data=p, REML=FALSE)

tab_model(delab_lme.2)

##Simple slopes:

pn.2 <- emtrends(delab_lme.2, var = "pn_pe.1",

at = list(pib_bin = c("0", "1")),

lmer.df = "satterthwaite")

summary(pn.2, infer=TRUE)

pn.3 <- emtrends(delab_lme.2, var = "c_curage",

at = list(pib_bin = c("0", "1")),

lmer.df = "satterthwaite")

summary(pn.3, infer=TRUE)

##ADD TAU:

delab_lme.1.1 <- lmer(ZPN_DelAB ~ c_curage + gender + readstn + (1|WRAPNo) + pn_pe.1*AT_bin, data=p, REML=FALSE)

tab_model(delab_lme.1.1)

##Simple slopes:

pn.4 <- emtrends(delab_lme.1.1, var = "pn_pe.1",

at = list(AT_bin = c("0", "1", "2", "3")),

lmer.df = "satterthwaite")

summary(pn.4, infer=TRUE)

##Add age:

delab_lme.1.2 <- lmer(ZPN_DelAB ~ c_curage + gender + readstn + (1|WRAPNo) + pn_pe.1*AT_bin +

c_curage*AT_bin, data=p, REML=FALSE)

tab_model(delab_lme.1.2)

pn.5 <- emtrends(delab_lme.1.2, var = "pn_pe.1",

at = list(AT_bin = c("0", "1", "2", "3")),

lmer.df = "satterthwaite")

summary(pn.5, infer=TRUE)

pn.6 <- emtrends(delab_lme.1.2, var = "c_curage",

at = list(AT_bin = c("0", "1", "2", "3")),

lmer.df = "satterthwaite")

summary(pn.6, infer=TRUE)

###################

##Plots:

##Aim 2 plots: LM & PN models:

wtot1_lme.1 <- lmer(ZwmsrTot ~ c_curage + gender + readstn + (c_curage|WRAPNo) + I(c_curage^2) +

lm_pe.1*pib_bin, data=p, REML=FALSE)

mod1.tb1 <- ggpredict(model=wtot1_lme.1, terms = c("lm_pe.1", "pib_bin"), group.terms = c("pib_bin"))

aim2.1 <- plot(mod1.tb1) +

ggtitle("LM Immediate") +

xlab(bquote(PE[nvis-1])) +

ylab("Predicted z-score") +

ylim(-0.55, 0.6) +

scale_color_manual(name="", labels = c("A-", "A+"), values=c("mediumseagreen", "midnightblue")) +

scale_fill_manual(values=c("mediumseagreen", "midnightblue"))+

theme(panel.grid.minor=element_blank(),

legend.position="bottom",

text=element_text(size=20),

plot.title=element_text(size=24))

aim2.1

##range -0.4 to 0.4

wtot2_lme.1 <- lmer(ZwmsrTot2 ~ c_curage + gender + readstn + (1|WRAPNo) + I(c_curage^2) + lm_pe.1*pib_bin, data=p, REML=FALSE)

mod2.tb1 <- ggpredict(model=wtot2_lme.1, terms = c("lm_pe.1", "pib_bin"), group.terms = c("pib_bin"))

aim2.2 <- plot(mod2.tb1) +

ggtitle("LM Delayed") +

xlab(bquote(PE[nvis-1])) +

ylab("Predicted z-score") +

ylim(-0.55, 0.6) +

scale_color_manual(name="", labels = c("A-", "A+"), values=c("mediumseagreen", "midnightblue")) +

scale_fill_manual(values=c("mediumseagreen", "midnightblue"))+

theme(panel.grid.minor=element_blank(),

legend.position="bottom",

text=element_text(size=20),

plot.title=element_text(size=24))

aim2.2

##-0.3, 0.6

immab_lme.1 <- lmer(ZPN_ImmAB ~ c_curage + gender + readstn + (1|WRAPNo) + pn_pe.1*pib_bin, data=p, REML=FALSE)

mod3.tb1 <- ggpredict(model=immab_lme.1, terms = c("pn_pe.1", "pib_bin"), group.terms = c("pib_bin"))

aim2.3 <- plot(mod3.tb1) +

ggtitle("PN Immediate") +

xlab(bquote(PE[nvis-1])) +

ylab("Predicted z-score") +

ylim(-0.5, 0.5) +

scale_color_manual(name="", labels = c("A-", "A+"), values=c("mediumseagreen", "midnightblue")) +

scale_fill_manual(values=c("mediumseagreen", "midnightblue"))+

theme(panel.grid.minor=element_blank(),

legend.position="bottom",

text=element_text(size=20),

plot.title=element_text(size=24))

aim2.3

##range -0.3, 0.3

delab_lme.1 <- lmer(ZPN_DelAB ~ c_curage + gender + readstn + (1|WRAPNo) + pn_pe.1*pib_bin, data=p, REML=FALSE)

mod4.tb1 <- ggpredict(model=delab_lme.1, terms = c("pn_pe.1", "pib_bin"), group.terms = c("pib_bin"))

aim2.4 <- plot(mod4.tb1) +

ggtitle("PN Delayed") +

xlab(bquote(PE[nvis-1])) +

ylab("Predicted z-score") +

ylim(-0.5, 0.5) +

scale_color_manual(name="", labels = c("A-", "A+"), values=c("mediumseagreen", "midnightblue")) +

scale_fill_manual(values=c("mediumseagreen", "midnightblue"))+

theme(panel.grid.minor=element_blank(),

legend.position="bottom",

text=element_text(size=20),

plot.title=element_text(size=24))

aim2.4

##-0.5, .3

##Pull all together:

aim2.fig <- ggarrange(aim2.3, aim2.4, aim2.1, aim2.2,

ncol=2, nrow=2, common.legend=TRUE, legend="bottom")

aim2.fig

##aim 2 w age LM and PN models:#############################################################

wtot1_lme.2 <- lmer(ZwmsrTot ~ c_curage + gender + readstn + (c_curage|WRAPNo) + I(c_curage^2) + lm_pe.1*pib_bin +

c_curage*pib_bin, data=p, REML=FALSE)

mod1.tb2 <- ggpredict(model=wtot1_lme.2, terms = c("lm_pe.1[all]", "pib_bin", "c_curage[-5.13, 0, 5.87]"),

group.terms = c("pib_bin"))

aim2s.1 <- plot(mod1.tb2) +

ggtitle("LM Immediate") +

xlab(bquote(PE[nvis-1])) +

ylab("Predicted z-score") +

ylim(-1.2, 1.6)+

scale_color_manual(name="", labels = c("A-", "A+"), values=c("mediumseagreen", "midnightblue")) +

scale_fill_manual(values=c("mediumseagreen", "midnightblue"))+

theme(panel.grid.minor=element_blank(),

legend.position="bottom",

text=element_text(size=20),

plot.title=element_text(size=24))

aim2s.1

wtot2_lme.2 <- lmer(ZwmsrTot2 ~ c_curage + gender + readstn + (1|WRAPNo) + I(c_curage^2) + lm_pe.1*pib_bin +

c_curage*pib_bin, data=p, REML=FALSE)

mod2.tb2 <- ggpredict(model=wtot2_lme.2, terms = c("lm_pe.1[all]", "pib_bin", "c_curage[-5.13, 0, 5.87]"),

group.terms = c("pib_bin"))

aim2s.2 <- plot(mod2.tb2) +

ggtitle("LM Delayed") +

xlab(bquote(PE[nvis-1])) +

ylab("Predicted z-score") +

ylim(-1.2, 1.6)+

scale_color_manual(name="", labels = c("A-", "A+"), values=c("mediumseagreen", "midnightblue")) +

scale_fill_manual(values=c("mediumseagreen", "midnightblue"))+

theme(panel.grid.minor=element_blank(),

legend.position="bottom",

text=element_text(size=20),

plot.title=element_text(size=24))

aim2s.2

immab_lme.2 <- lmer(ZPN_ImmAB ~ c_curage + gender + readstn + (1|WRAPNo) + pn_pe.1*pib_bin +

pib_bin*c_curage, data=p, REML=FALSE)

mod3.tb2 <- ggpredict(model=immab_lme.2, terms = c("pn_pe.1[all]", "pib_bin", "c_curage[-5.13, 0, 5.87]"),

group.terms = c("pib_bin"))

aim2s.3 <- plot(mod3.tb2) +

ggtitle("PN Immediate") +

xlab(bquote(PE[nvis-1])) +

ylab("Predicted z-score") +

ylim(-1, 1) +

scale_color_manual(name="", labels = c("A-", "A+"), values=c("mediumseagreen", "midnightblue")) +

scale_fill_manual(values=c("mediumseagreen", "midnightblue"))+

theme(panel.grid.minor=element_blank(),

legend.position="bottom",

text=element_text(size=20),

plot.title=element_text(size=24))

aim2s.3

delab_lme.2 <- lmer(ZPN_DelAB ~ c_curage + gender + readstn + (1|WRAPNo) + pn_pe.1*pib_bin +

c_curage*pib_bin, data=p, REML=FALSE)

mod4.tb2 <- ggpredict(model=delab_lme.2, terms = c("pn_pe.1[all]", "pib_bin", "c_curage[-5.13, 0, 5.87]"),

group.terms = c("pib_bin"))

aim2s.4 <- plot(mod4.tb2) +

ggtitle("PN Delayed") +

xlab(bquote(PE[nvis-1])) +

ylab("Predicted z-score") +

ylim(-1, 1) +

scale_color_manual(name="", labels = c("A-", "A+"), values=c("mediumseagreen", "midnightblue")) +

scale_fill_manual(values=c("mediumseagreen", "midnightblue"))+

theme(panel.grid.minor=element_blank(),

legend.position="bottom",

text=element_text(size=20),

plot.title=element_text(size=24))

aim2s.4

summary(p$curage)

##Pull all together:

aim2s.fig <- ggarrange( aim2s.3, aim2s.4, aim2s.1, aim2s.2,

ncol=2, nrow=2, common.legend=TRUE, legend="bottom")

aim2s.fig

##Aim 3:#########################################

ani_lme.1.1 <- lmer(Zanim ~ c_curage + gender + readstn + (1|WRAPNo) + an_pe.1*AT_bin, data=p, REML=FALSE)

mod5.tb3 <- ggpredict(model=ani_lme.1.1, terms = c("an_pe.1", "AT_bin"),

group.terms = c("AT_bin"))

aim3.1 <- plot(mod5.tb3) +

ggtitle("Animal Fluency") +

xlab(bquote(PE[nvis-1])) +

ylab("Predicted z-score") +

ylim(-.8, 1.2) +

scale_color_manual(name="", labels = c("A-T-", "A+T-", "A+T+", "A-T+"),

values=c("#0072B2", "#E69F00", "#CC79A7", "#009E73")) +

scale_fill_manual(values=c("#0072B2", "#E69F00", "#CC79A7", "#009E73"))+

theme(panel.grid.minor=element_blank(),

legend.position="bottom",

text=element_text(size=20),

plot.title=element_text(size=24))

aim3.1

cfl_lme.1.1 <- lmer(Zflucfl ~ c_curage + gender + readstn + (1|WRAPNo) + I(c_curage^2) + cfl_pe.1*AT_bin, data=p, REML=FALSE)

mod6.tb3 <- ggpredict(model=cfl_lme.1.1, terms = c("cfl_pe.1", "AT_bin"),

group.terms = c("AT_bin"))

aim3.2 <- plot(mod6.tb3) +

ggtitle("Letter Fluency") +

xlab(bquote(PE[nvis-1])) +

ylim(-.8, 1.2) +

ylab("Predicted z-score") +

scale_color_manual(name="", labels = c("A-T-", "A+T-", "A+T+", "A-T+"),

values=c("#0072B2", "#E69F00", "#CC79A7", "#009E73")) +

scale_fill_manual(values=c("#0072B2", "#E69F00", "#CC79A7", "#009E73"))+

theme(panel.grid.minor=element_blank(),

legend.position="bottom",

text=element_text(size=20),

plot.title=element_text(size=24))

aim3.2

wtot1_lme.1.1 <- lmer(ZwmsrTot ~ c_curage + gender + readstn + (c_curage|WRAPNo) + I(c_curage^2) +

lm_pe.1*AT_bin, data=p, REML=FALSE)

mod1.tb3 <- ggpredict(model=wtot1_lme.1.1, terms = c("lm_pe.1", "AT_bin"),

group.terms = c("AT_bin"))

plot(mod1.tb3)

aim3.3 <- plot(mod1.tb3) +

ggtitle("LM Immediate") +

xlab(bquote(PE[nvis-1])) +

ylab("Predicted z-score") +

ylim(-1.2, 1) +

scale_color_manual(name="", labels = c("A-T-", "A+T-", "A+T+", "A-T+"),

values=c("#0072B2", "#E69F00", "#CC79A7", "#009E73")) +

scale_fill_manual(values=c("#0072B2", "#E69F00", "#CC79A7", "#009E73"))+

theme(panel.grid.minor=element_blank(),

legend.position="bottom",

text=element_text(size=20),

plot.title=element_text(size=24))

aim3.3

wtot_lme.1.1 <- lmer(ZwmsrTot2 ~ c_curage + gender + readstn + (1|WRAPNo) + I(c_curage^2)

+ lm_pe.1*AT_bin, data=p, REML=FALSE)

mod2.tb3 <- ggpredict(model=wtot_lme.1.1, terms = c("lm_pe.1", "AT_bin"),

group.terms = c("AT_bin"))

aim3.4 <- plot(mod2.tb3) +

ggtitle("LM Delayed") +

xlab(bquote(PE[nvis-1])) +

ylab("Predicted z-score") +

ylim(-1.2, 1) +

scale_color_manual(name="", labels = c("A-T-", "A+T-", "A+T+", "A-T+"),

values=c("#0072B2", "#E69F00", "#CC79A7", "#009E73")) +

scale_fill_manual(values=c("#0072B2", "#E69F00", "#CC79A7", "#009E73"))+

theme(panel.grid.minor=element_blank(),

legend.position="bottom",

text=element_text(size=20),

plot.title=element_text(size=24))

aim3.4

immab_lme.1.1 <- lmer(ZPN_ImmAB ~ c_curage + gender + readstn + (1|WRAPNo) + pn_pe.1*AT_bin, data=p, REML=FALSE)

mod3.tb3 <- ggpredict(model=immab_lme.1.1, terms = c("pn_pe.1", "AT_bin"),

group.terms = c("AT_bin"))

aim3.5 <- plot(mod3.tb3) +

ggtitle("PN Immediate") +

xlab(bquote(PE[nvis-1])) +

ylab("Predicted z-score") +

ylim(-1.2, 0.6) +

scale_color_manual(name="", labels = c("A-T-", "A+T-", "A+T+", "A-T+"),

values=c("#0072B2", "#E69F00", "#CC79A7", "#009E73")) +

scale_fill_manual(values=c("#0072B2", "#E69F00", "#CC79A7", "#009E73"))+

theme(panel.grid.minor=element_blank(),

legend.position="bottom",

text=element_text(size=20),

plot.title=element_text(size=24))

aim3.5

delab_lme.1.1 <- lmer(ZPN_DelAB ~ c_curage + gender + readstn + (1|WRAPNo) + pn_pe.1*AT_bin, data=p, REML=FALSE)

mod4.tb3 <- ggpredict(model=delab_lme.1.1, terms = c("pn_pe.1", "AT_bin"),

group.terms = c("AT_bin"))

aim3.6 <- plot(mod4.tb3) +

ggtitle("PN Delayed") +

xlab(bquote(PE[nvis-1])) +

ylab("Predicted z-score") +

ylim(-1.2, 0.6) +

scale_color_manual(name="", labels = c("A-T-", "A+T-", "A+T+", "A-T+"),

values=c("#0072B2", "#E69F00", "#CC79A7", "#009E73")) +

scale_fill_manual(values=c("#0072B2", "#E69F00", "#CC79A7", "#009E73"))+

theme(panel.grid.minor=element_blank(),

legend.position="bottom",

text=element_text(size=20),

plot.title=element_text(size=24))

aim3.6

##Pull all together:

aim3.fig <- ggarrange(aim3.5, aim3.6, aim3.3, aim3.4, aim3.1, aim3.2,

ncol=2, nrow=3, common.legend=TRUE, legend="bottom")

aim3.fig

##aim 3 + age:

ani_lme.1.2 <- lmer(Zanim ~ c_curage + gender + readstn + (1|WRAPNo) + an_pe.1*AT_bin + c_curage*AT_bin, data=p, REML=FALSE)

mod5.tb4 <- ggpredict(model=ani_lme.1.2, terms = c("an_pe.1", "AT_bin", "c_curage[-5.13, 0, 5.87]"),

group.terms = c("AT_bin"))

aim3s.1 <- plot(mod5.tb4) +

ggtitle("Animal Fluency") +

xlab(bquote(PE[nvis-1])) +

ylab("Predicted z-score") +

scale_color_manual(name="", labels = c("A-T-", "A+T-", "A+T+", "A-T+"),

values=c("#0072B2", "#E69F00", "#CC79A7", "#009E73")) +

scale_fill_manual(values=c("#0072B2", "#E69F00", "#CC79A7", "#009E73"))+

theme(panel.grid.minor=element_blank(),

legend.position="bottom",

text=element_text(size=20),

plot.title=element_text(size=24))

aim3s.1

cfl_lme.1.2 <- lmer(Zflucfl ~ c_curage + gender + readstn + (1|WRAPNo) + I(c_curage^2) + cfl_pe.1*AT_bin +

c_curage*AT_bin, data=p, REML=FALSE)

mod6.tb4 <- ggpredict(model=cfl_lme.1.2, terms = c("cfl_pe.1", "AT_bin", "c_curage[-5.13, 0, 5.87]"),

group.terms = c("AT_bin"))

aim3s.2 <- plot(mod6.tb4) +

ggtitle("Letter Fluency") +

xlab(bquote(PE[nvis-1])) +

ylab("Predicted z-score") +

scale_color_manual(name="", labels = c("A-T-", "A+T-", "A+T+", "A-T+"),

values=c("#0072B2", "#E69F00", "#CC79A7", "#009E73")) +

scale_fill_manual(values=c("#0072B2", "#E69F00", "#CC79A7", "#009E73"))+

theme(panel.grid.minor=element_blank(),

legend.position="bottom",

text=element_text(size=20),

plot.title=element_text(size=24))

aim3s.2

wtot1_lme.1.2 <- lmer(ZwmsrTot ~ c_curage + gender + readstn + (c_curage|WRAPNo) + I(c_curage^2) + lm_pe.1*AT_bin +c_curage*AT_bin

, data=p, REML=FALSE)

mod1.tb4 <- ggpredict(model=wtot1_lme.1.2, terms = c("lm_pe.1", "AT_bin", "c_curage[-5.13, 0, 5.87]"),

group.terms = c("AT_bin"))

aim3s.3 <- plot(mod1.tb4) +

ggtitle("LM Immediate") +

xlab(bquote(PE[nvis-1])) +

ylab("Predicted z-score") +

scale_color_manual(name="", labels = c("A-T-", "A+T-", "A+T+", "A-T+"),

values=c("#0072B2", "#E69F00", "#CC79A7", "#009E73")) +

scale_fill_manual(values=c("#0072B2", "#E69F00", "#CC79A7", "#009E73"))+

theme(panel.grid.minor=element_blank(),

legend.position="bottom",

text=element_text(size=20),

plot.title=element_text(size=24))

aim3s.3

wtot_lme.1.2 <- lmer(ZwmsrTot2 ~ c_curage + gender + readstn + (1|WRAPNo) + I(c_curage^2)

+ lm_pe.1*AT_bin + c_curage*AT_bin, data=p, REML=FALSE)

mod2.tb4 <- ggpredict(model=wtot_lme.1.2, terms = c("lm_pe.1", "AT_bin", "c_curage[-5.13, 0, 5.87]"),

group.terms = c("AT_bin"))

aim3s.4 <- plot(mod2.tb4) +

ggtitle("LM Delayed") +

xlab(bquote(PE[nvis-1])) +

ylab("Predicted z-score") +

scale_color_manual(name="", labels = c("A-T-", "A+T-", "A+T+", "A-T+"),

values=c("#0072B2", "#E69F00", "#CC79A7", "#009E73")) +

scale_fill_manual(values=c("#0072B2", "#E69F00", "#CC79A7", "#009E73"))+

theme(panel.grid.minor=element_blank(),

legend.position="bottom",

text=element_text(size=20),

plot.title=element_text(size=24))

aim3s.4

immab_lme.1.2 <- lmer(ZPN_ImmAB ~ c_curage + gender + readstn + (1|WRAPNo) + pn_pe.1*AT_bin +

c_curage*AT_bin, data=p, REML=FALSE)

mod3.tb4 <- ggpredict(model=immab_lme.1.2, terms = c("pn_pe.1", "AT_bin", "c_curage[-5.13, 0, 5.87]"),

group.terms = c("AT_bin"))

aim3s.5 <- plot(mod3.tb4) +

ggtitle("PN Immediate") +

xlab(bquote(PE[nvis-1])) +

ylab("Predicted z-score") +

scale_color_manual(name="", labels = c("A-T-", "A+T-", "A+T+", "A-T+"),

values=c("#0072B2", "#E69F00", "#CC79A7", "#009E73")) +

scale_fill_manual(values=c("#0072B2", "#E69F00", "#CC79A7", "#009E73"))+

theme(panel.grid.minor=element_blank(),

legend.position="bottom",

text=element_text(size=20),

plot.title=element_text(size=24))

aim3s.5

delab_lme.1.2 <- lmer(ZPN_DelAB ~ c_curage + gender + readstn + (1|WRAPNo) + pn_pe.1*AT_bin +

c_curage*AT_bin, data=p, REML=FALSE)

mod4.tb4 <- ggpredict(model=delab_lme.1.2, terms = c("pn_pe.1", "AT_bin", "c_curage[-5.13, 0, 5.87]"),

group.terms = c("AT_bin"))

aim3s.6 <- plot(mod4.tb4) +

ggtitle("PN Delayed") +

xlab(bquote(PE[nvis-1])) +

ylab("Predicted z-score") +

scale_color_manual(name="", labels = c("A-T-", "A+T-", "A+T+", "A-T+"),

values=c("#0072B2", "#E69F00", "#CC79A7", "#009E73")) +

scale_fill_manual(values=c("#0072B2", "#E69F00", "#CC79A7", "#009E73"))+

theme(panel.grid.minor=element_blank(),

legend.position="bottom",

text=element_text(size=20),

plot.title=element_text(size=24))

aim3s.6

##Pull all together:

aim3s.fig <- ggarrange(aim3s.5, aim3s.6, aim3s.3, aim3s.4, aim3s.1, aim3s.2,

ncol=2, nrow=3, common.legend=TRUE, legend="bottom")

aim3s.fig
